# Prevalence and factors associated with fear of childbirth: national online survey of Brazilian adults^*^

**DOI:** 10.1590/1518-8345.7617.4897

**Published:** 2026-08-21

**Authors:** Andrezza Belluomini Castro, Rafaela Aparecida Prata, Marla Andréia Garcia de Avila, Milena Temer Jamas

**Affiliations:** 1 Universidade Estadual Paulista, Departamento de Enfermagem, Botucatu, SP, Brazil.

**Keywords:** Obstetric Nursing, Fear, Childbirth, Internet, Health Education, Pregnant Person.

## Abstract

**Objective:**

to identify the prevalence and factors associated with fear of childbirth in Brazilian adults.

**Method:**

cross-sectional study with 528 participants aged between 18 and 58 years. Data were collected via an online questionnaire that addressed sociodemographic and behavioral characteristics, as well as information related to health, obstetric history, and fear of childbirth, using the Brazilian version of the Childbirth Fear Prior to Pregnancy instrument. The variables were analyzed and adjusted by multiple linear regression models, assuming that the residuals follow a normal distribution.

**Results:**

the prevalence of fear of childbirth was 28.4% (n=133) for low, 19.5% (n=103) for moderate, and 26.9% (n=142) for high fear of childbirth. Multivariate analyses showed that lower fear of childbirth scores were associated with younger age (p=0.002) and female gender (p<0.000). On the other hand, individuals with previous pregnancies had higher CPFF scores (p=0.005).

**Conclusion:**

the prevalence of fear of childbirth was 71.6% (n=378), distributed between low (25.2%), moderate (19.5%), and high fear (26.9%). Factors such as older age and female gender were associated with lower fear scores, while previous pregnancy was linked to a higher level of fear. These results suggest that maternal experience may influence future decisions about the choice of childbirth or impact the perception of other people in their social circle.

## Highlights

**(1)** Higher scores for fear of childbirth were associated with female gender. **(2)** Prevalence of fear of childbirth in the population who wish to have children. **(3)** The younger population shows more fear of childbirth. **(4)** Greater association with fear of childbirth in women with previous experience.

## Introduction

Fear of childbirth is characterized by feelings of insecurity and anxiety before, during, or after birth in relation to the labor process and the moment of delivery, with varying degrees of severity^1^ ^-^ ^2^. This feeling may result from bad reports or experiences in previous childbirths caused by hostile treatment by the team, feelings of abandonment, or lack of participation in decisions regarding care^3^ ^-^ ^4^.

Intense fear of childbirth is a multifaceted issue that extends far beyond the obstetric event itself, significantly impacting women’s mental health and emotional well-being in the long term. As evidenced by the literature, the complexity of childbirth, which mobilizes a range of contradictory feelings such as anxiety, insecurity, and stress, already points to the relevance of the psychological aspects involved^5^.

Scientific evidence indicates that sociocultural factors play a significant role in triggering fear of childbirth. Cultural narratives, transmitted by family and friends, that associate vaginal delivery with pain and suffering contribute to women developing anxiety, insecurity, and intensified fears. This results in an underestimation of their own physiological capacity to give birth, despite the millennial history of natural childbirth without medicalization. The great challenge in obstetric care is, therefore, to give pregnant women back control over their bodies and thoughts, promoting this confidence throughout the entire prenatal period, and not just in the hours leading up to childbirth^6^.

This deep apprehension can lead to a series of consequences that manifest in various ways in women’s lives. In addition to the already known physiological effects-such as prolonged dilation, increased risk of preeclampsia, premature childbirth, and emergency cesarean section-fear of childbirth is intrinsically linked to negative mental health outcomes and quality of life. It can lead to the development of postpartum depression, affect breastfeeding rates, and even result in a greater need for newborn admission to intensive care units^7^ ^-^ ^8^.

This study identified that the first pregnancy, lower educational level, natural conception, and preference for cesarean section are associated with a higher level of fear. In addition, comorbidities such as high myopia and urinary tract diseases were identified as independent predictors of severe fear. Recognizing the complexity of fear of childbirth is crucial for healthcare professionals who perform obstetric screening in prenatal care, especially in at-risk groups. This allows them to offer personalized counseling and support, promoting informed childbirth choices and contributing to maternal psychological well-being and better obstetric outcomes^9^.

Sweden is one of the few countries where fear of childbirth is recognized as a significant problem and where counseling services are available to most pregnant women. A 2006 survey of 2,262 Swedish women indicated that the prevalence of fear of childbirth was around 10%. The women were recruited at their first prenatal clinic visit during three predetermined weeks spread over a year. The study revealed that fear of childbirth was associated with an increased rate of elective cesarean sections and an “acceptable” childbirth experience in women who underwent counseling^4^.

A study conducted in Croatia involving young, non-pregnant female university students studying health sciences, social sciences, and humanities showed that 25.9% of students reported significant fear of childbirth, with health science students reporting lower levels of fear compared to students in other programs, and they had received more information about childbirth in the family context compared to the learning process. It is believed that the highest level of fear of childbirth could be predicted by identifying factors such as sensitivity to anxiety, anxiety traits, expectation of pain during childbirth, and sources of knowledge about childbirth^10^.

Fear of childbirth negatively affects women in several ways, which can trigger increased use of labor analgesia, emotional imbalance, prolonged labor, the choice of elective cesarean section, difficulty bonding with the baby, and postpartum depression^11^ ^-^ ^14^. Additionally, it can impact harmony in family and partner relationships. There is substantial evidence indicating that high levels of fear of childbirth lead to post-traumatic stress disorder, postpartum anxiety, depression, and psychosis, and even increase the need for long-term psychiatric care^15^ ^-^ ^16^ ^).^

Growing evidence suggests that fear of childbirth is not exclusive to women; expectant fathers may also experience significant anxieties related to birth, although their concerns and manifestations may differ. Including the male perspective in research on fear of childbirth is essential, not only to understand the paternal experience itself, but also because the father’s well-being can impact his partner, family dynamics, and the transition to parenthood^17^. Ignoring the fear of childbirth in men means leaving a portion of the population without the necessary support and understanding during a period of vulnerability.

The adoption of measures to assess and intervene in childbirth contributes to demystifying fears and misconceptions, improving the well-being and quality of life of young people, and reinforcing international efforts to reduce elective cesarean section rates and medicalization. It is important to assess and intervene in advance in non-pregnant populations, since it is during pregnancy that fears become more intense^18^.

In this scenario, nursing plays a central and irreplaceable role. Nursing professionals, with their proximity and holistic approach to perinatal care, are in a privileged position to facilitate demystifying childbirth and reducing the fear associated with it. Through active listening, comprehensive perinatal education, and the implementation of evidence-based personalized care practices, nursing can empower both women and men. This involves deconstructing myths, providing clear and realistic information about the birth process, discussing pain management options, promoting the couple’s protagonism, and creating an environment of trust and safety^19^ ^-^ ^21^.

A qualitative study^22^ investigated women’s perceptions of midwives’ counseling regarding fear of childbirth. The results revealed an improvement in women’s confidence in childbirth after acquiring information and knowledge. Participants reported feeling calmer and more prepared, which strengthened their ability to cope with the uncertainty of the birthing process, positively impacting the experience of giving birth. Additionally, the feeling of security provided by professional support during childbirth contributed to women feeling empowered. This positive experience, in turn, reinforced self-confidence for future births, and the fear of childbirth was perceived as reduced or controllable.

Despite their proven effectiveness in reducing pain and shortening labor and delivery time, and their low cost and ease of application, non-pharmacological methods such as heat therapy, massage, aromatherapy, acupressure, dance, and Swiss ball exercises are still not widely known. These approaches are also associated with lower use of pain medication. However, it is in the context of childbirth, with the presence and involvement of obstetric nurses, that these methods gain greater visibility and are more widely used^23^ ^-^ ^24^. Nursing plays a crucial role as a facilitator in demystifying childbirth and reducing fear, actively promoting these personalized and evidence-based care practices, which empower women and contribute to a more positive and less medicalized childbirth experience.

It is imperative to investigate the factors associated with fear of childbirth, broadening the traditional scope to include the male population and considering assessment in the stages preceding pregnancy, particularly in the Brazilian context. A thorough understanding of these factors is essential to support the development of more effective, inclusive, and culturally sensitive intervention strategies, especially those promoted by nursing, aiming to improve perinatal health and birth experience for all involved. Thus, this study aimed to identify the prevalence and factors associated with fear of childbirth in Brazilian adults.

## Method

### Study design

This is a cross-sectional and analytical study conducted using an online survey of adults over 18 years of age, Brazilians from the five macro-regions of the country, guided by the Strengthening the Reporting of Observational Studies in Epidemiology (STROBE) guideline^25^, carried out from January to April 2022 using a digital form provided by the Google Forms tool.

### Sample definition 

The study included men and women aged 18 or older, regardless of marital status, who intended to have children in the future. Participants who did not respond to the instrument during the data collection period and those whose forms lacked complete responses to all questions were excluded.

The study included men and women aged 18 or older, regardless of marital status, who intended to have children in the future. Participants who did not respond to the instrument during the data collection period and those whose forms lacked complete responses to all questions were excluded.

Given the target population, consisting of adults over 18 years of age who intend to have children in the future, residing in any region of the country, the sample is classified as non-probabilistic and intentional. It is prudent to consider that the non-probabilistic intentional sampling plan adopted for 528 participants may introduce systematic selection errors that influence estimates of the associations between the investigated factors and the fear of childbirth score, especially if the aim is to infer the Brazilian population.

However, to estimate its size, it was assumed that the intention was to understand a process and not to infer a specifically defined population in time and space. Therefore, to calculate the minimum number of participants (not the minimum size of a representative sample of a target population), a prevalence of 0.50, a significance level of 5%, and an accuracy of 3% were assumed.

### Study variables

The independent variables were: sociodemographic data such as age (in years); gender (male; female; prefer not to identify); self-reported skin color (white; black; brown; indigenous; yellow); education (no education; incomplete elementary school; complete elementary school; incomplete high school; complete high school; higher education; postgraduate); origin (North; Northeast; South; Southeast; Midwest); health professional (yes or no); marital status (with partner or without partner). Obstetric variables: has children (yes or no); type of childbirth in last pregnancy (normal; cesarean); had a negative experience in previous pregnancy (yes; no); Behavioral variables: experiences of friends or family members influenced the type of delivery chosen (yes; no); source of information for the study population: where to find information about childbirth (videos/internet; printed material; relatives and friends; healthcare professional; scientific articles; I do not seek information about childbirth). In this study, the Childbirth Fear Prior to Pregnancy (CFPP) scale was considered as a dependent variable as an instrument for assessing fear of childbirth, as it allows for the assessment of fear before conception in men and women. It is recognized for its accessible language and ease of application, which help develop targeted interventions. Its dimensions address fear of childbirth pain, fear of complications related to the baby, and fear of bodily harm^18^.

### Data collection

For this study, researchers developed a virtual data collection tool using the Google Forms platform. This questionnaire consisted of 26 closed-ended questions to gather information on the participants’ sociodemographic, obstetric, and behavioral characteristics (independent variables). 

The Brazilian online version of the CFPP was adopted as the instrument for measuring the dependent variable, as it is the only instrument validated for the Brazilian population for assessing fear of childbirth in men and women^26^. Another relevant point is its adequate psychometric properties, including clear, easy-to-understand language, which facilitates its application. These qualities make the CFPP a valuable tool not only for research but also for the development of effective interventions to reduce fear of childbirth^18^.

The Brazilian online version of the CFPP is a short and simple, one-dimensional measurement tool consisting of 10 items with 5 response options ranging from (1) strongly disagree, (2) disagree, (3) partially disagree, (4) agree, and (5) strongly agree, arranged vertically. The final score is obtained by adding up the scores for the 10 items, with a minimum score of 10 and a maximum score of 50^26^.

Data collection was performed through an online survey, following the recommendations of the CHERRIES (Checklist for Reporting Results of Internet E-Surveys) list^27^, which made it possible, at a time that required social distancing, to achieve a diverse sample via the internet, covering individuals in different geographic regions of Brazil and in an immediate manner^28^.

Data collection was carried out by the researchers and three undergraduates who collaborated on the study. Participants were recruited through the dissemination of a digital form, via Google Forms, across various online channels, including emails, social networks (Facebook and Instagram), and personal contacts via WhatsApp. This process expanded through a multiplier effect called “snowballing” or link tracking sampling. Snowball sampling is a non-probabilistic technique that generates a multiplier effect in data collection, like the growth of a snowball. The process begins with identifying one or more “key informants”. These, in turn, indicate new participants from their contact networks who meet the research criteria, creating an exponential chain of references. This approach is particularly effective for reaching hard-to-reach groups or those dealing with sensitive issues, as it leverages existing connections between individuals^29^.

### Data analysis

The collected data were transferred and organized using Microsoft Office Excel. Statistical analyses were performed using IBM Statistical Package for the Social Sciences (SPSS) software (version 21).

First, exploratory data analysis was performed to identify and address outliers and to determine the mechanism generating missing values. After initial evaluation, a bivariate analysis was performed to investigate the isolated association of each independent variable with the CFPP scale score using simple linear regressions with a normal probability distribution response (which accommodates numerical outcomes with at least a reasonable degree of symmetry).

The cut-off p < 0.20 is used to avoid omitting a variable that plays an important and well-established role in a social determinants model or in the pathogenesis of the outcome solely because of a weaker association. In other words, it prioritizes health paradigms over statistical criteria. In the multiple regression model, associations were considered statistically significant if p < 0.05.

### Ethical aspects

All activities were carried out after approval by the Research Ethics Committee of the Botucatu School of Medicine, under opinion No. 4,836,471, in accordance with National Health Council Resolution No. 510/2016. Agreement to participate in the study was recorded using a digital Free and Informed Consent Form (FICF)^30^.

## Results

A total of 671 individuals agreed to participate in the study. However, 143 participants were excluded for not meeting the inclusion criteria, leaving a final sample of 528 participants from the five macro-regions of Brazil.

Of the participants, 81.6% (n=431) were female, with a median age of 24 (minimum of 18 and maximum of 58), 69.3% (n=366) self-identified as white or Asian, 75.8% (n=400) had at least a high school education, 50.9% (n=269) were health professionals, and 61% (n=322) reported having a partner. The Southeast region was the most represented, with 62.1% (n=328) of participants, as shown in Table 1.

**Table 1:** Sociodemographic characteristics of participants (n = 528). Brazil, 2024

Variables	Total	
	n*	%^†^
Gender		
Female	431	81.6
Male	96	18.2
Not identified	1	0.2
**Race/Color**		
White/Yellow	366	69.3
Black/Brown/Indigenous	162	30.7
**Education**		
Incomplete elementary and secondary education	128	24.2
High school, Undergraduate, and Graduate studies	400	75.8
**Marital status**		
With partner	322	61.0
Without a partner	206	39.0
**Region**		
Southeast	328	62.1
Midwest	83	15.7
Northeast	57	10.8
South	35	6.6
North	25	4.7
**Healthcare professional**		
Yes	269	50.9
No	259	49.1

*n = Number of participants; ^†^% = Percentage

Of the 95 participants who reported having children, 29.4% (n=28) had experience with normal childbirth or had experienced it with their partners. In addition, 33 (6.3%) of the participants reported negative experiences in their last pregnancy, and 71.6% (n=378) reported fear of childbirth. The search for information about childbirth was reported by 74.6% (n=394), with 26.3% (n=139) seeking this information mainly from health professionals and 22.2% (n=117) on the internet and in videos, as shown in Table 2.

**Table 2:** Obstetric characteristics and perceptions about childbirth and sources of information among adults. Brazil, 2024

Variables	n*	%^†^
Have children (n=528)	95	18.0
Vaginal delivery in the last pregnancy (Yes) (n=95)	28	29.5
Negative experience during last pregnancy (Yes) (n=95)	33	34.7
Experiences of friends and family influence the choice of delivery method (Yes) (n=528)	123	23.3
Fear of childbirth (Yes) (n=528)	378	71.6
Search for information about childbirth (Yes) (n=528)	394	74.6
**Places where they seek information**		
Healthcare professional	139	26.3
Videos/internet	117	22.2
Scientific articles	79	15.0
Relatives and friends	54	10.2
Printed material	05	0.9

*n = Number of participants; ^†^% = Percentage

Table 3 shows the participants’ scores for fear of childbirth, according to the CFPP classification. The prevalence of fear of childbirth was found to be 71.6% (n=378), with 25.2% (n=133) having low fear, 19.5% (n=103) moderate fear, and 26.9% (n=142) high fear of childbirth.

**Table 3:** Classification of fear of childbirth in adults according to CFPP* (n = 528). Brazil, 2024

Classification	CFPP* score	n^†^	%^‡^
No fear	10 ≤ x ≤ 28	150	28.4
Low fear	29 ≤ x ≤ 33	133	25.2
Moderate fear	34 ≤ x ≤ 37	103	19.5
High fear	38 ≤ x ≤ 50	142	26.9

*Version of the Childbirth Fear Prior to Pregnancy scale with scores ranging from 10 to 50; ^†^n = Number of participants; ^‡^% = Percentage

According to the results presented in Table 4, the bivariate analysis investigated the association between independent variables and the CFPP scale score. The variables age (p < 0.001), female gender (p < 0.001), not being a health professional (p = 0.027), residing in the Southeast (p = 0.130) and Northeast (p = 0.150) regions, marital status with a partner (p = 0.137), not having children (0.051), having more than one pregnancy (p = 0.001), experience of friends/family choosing the type of delivery (p=0.017), seeking information in scientific articles (p=0.035), relatives and friends (p=0.027), health professionals (p=0.007), and videos/internet (p=0.007).

**Table 4:** Bivariate association between sociodemographic, obstetric, behavioral variables, and the CFPP* score (n = 528). Brazil, 2024

Variables	b^†^	95% ^‡^CI		*p* ^*§*^
		Lower Limit	Upper Limit	
Age	-0.17	-.25	-.08	**0.000**
Gender				
Male	3.80	-10.95	18.56	0.614
Female	-3.48	-5.14	-1.81	**0.000**
Another classification^||^				
Race or Color				
White/Yellow	0.32	-1.09	1.74	0.654
Black/Brown/Indigenous^¶^				
Region				
Midwest	-2.35	-6.23	1.54	0.236
Southeast	2.46	-.72	5.65	**0.130**
South	1.18	-1.81	4.17	0.441
Northeast	1.94	-.70	4.58	**0.150**
North				
Not a healthcare professional	-1.47	-2.77	-0.17	**0.027**
Labor	-1.89	-4.85	1.06	0.210
Minimum education level: high school	0.23	-1.29	1.75	0.768
Marital status: with partner	-1.01	-2.35	0.32	**0.137**
No children	-1.69	-3.38	0.00	**0.051**
More than one pregnancy	4.94	2.06	7.82	**0.001**
Negative experience during last pregnancy	-0.22	-2.91	2.47	0.871
Experience of friends/family choice of type of delivery	1.08	-.46	2.62	**0.170**
Seeking information about delivery	-0.30	-1.80	1.19	0.691
Places where information is sought				
Scientific articles	-1.95	-3.77	-0.13	**0.035**
Printed materials	-1.39	-8.11	5.34	0.687
Relatives, friends	2.42	.27	4.56	**0.027**
Health professionals	-2.01	-3.48	-.54	**0.007**
Video/internet	2.15	.59	3.71	**0.007**

*Version of the Childbirth Fear Prior to Pregnancy scale; ^†^Intercept; ^‡^Confidence interval; ^§^Bivariate analysis by simple linear regression; ^||^Reference for gender; ^¶^Reference for color/race; **Reference for regions in Brazil

A multivariate analysis was performed using multiple linear regression, with the total CFPP scale score as the dependent variable (scores ranging from 10 to 50). The results showed that older age was associated with lower scores on the scale, indicating lower fear of childbirth (b = -0.17; 95% CI: -0.25 to -0.08; p < 0.001). In addition, men had an average score 3.6 points lower than women, demonstrating less fear of childbirth (b = -3.48; 95% CI -5.14 to -1.81; p < 0.001). On the other hand, participants with previous pregnancy experience had higher scores than those who reported no childbirth experience; they had an average of 4.5 points higher, indicating greater fear of childbirth (b = 4.52; 95% CI 1.35 to 7.68; p = 0.005) (Table 5).

**Table 5:** Multiple linear regression between sociodemographic, obstetric, and behavioral variables and the total CFPP* scale score (n = 528). Brazil, 2024

Variables	b^†^	95% ^‡^CI		*p* ^*§*^
		Lower Limit	Upper Limit	
Age	-0.16	-.26	-.06	**0.002**
Gender				
Male	1.87	-12.22	15.96	0.795
Female	-3.68	-5.32	-2.04	**0.000**
Other classification^||^				
Region
Midwest	-1.89	-5.65	1.87	0.324
Southeast	0.97	-2.09	4.03	0.534
South	0.77	-2.09	3.64	0.597
Northeast	1.17	-1.37	3.71	0.365
North^¶^				
Not a healthcare professional	-1.22	-2.54	0.11	0.072
Marital status with partner	0.13	-1.25	1.50	0.855
No children	1.26	-.98	3.50	0.271
Experience of friends/family in the choice of type of delivery	4.52	1.35	7.68	**0.005**
Seeking information from relatives, friends	1.45	-.07	2.97	0.062
Seeking information in scientific articles	1.58	-0.76	3.93	0.186
Seeking information from health professionals	1.60	-3.70	.50	0.134
Seeking information from videos/the Internet	-1.43	-3.18	.33	0.110

*Version of the Childbirth Fear Prior to Pregnancy scale; ^†^Intercept; ^‡^Confidence interval; ^§^Bivariate analysis by multiple linear regression; ^||^Reference for gender; ^¶^Reference for regions in Brazil

## Discussion

The highlight of this study was to seek evidence, using an instrument appropriate to the Brazilian context, that could help identify factors associated with fear of normal childbirth in the Brazilian population, including men and women, thereby providing support for the development of educational materials and policy actions on the subject.

Tocophobia, or intense fear of childbirth, affects approximately 14% of women globally^11^. When analyzing prevalence across countries, a significant variation is observed. In the United States, for example, the condition is more prevalent, affecting 26.9% of pregnant women^31^. In countries such as Australia and Finland, the rate of fear of childbirth is between 20% and 25%^32^ ^-^ ^33^. These data highlight the importance of understanding and addressing tocophobia, considering its cultural and geographical particularities.

A qualitative study conducted in the United Kingdom, involving 10 pregnant women and 13 midwives, identified important factors that influence fear of childbirth, such as fear of not being able to cope with the unpredictable; fear that the baby or the pregnant woman herself will be injured during childbirth; fear of not being able to give birth; fear of not knowing how to deal with pain; fear of not having autonomy in decision-making; fear of interventions; fear of abandonment; and fear of losing control. In this study, when asked when questions about fear of childbirth should be asked, most respondents responded that it should be as early as possible. It emphasized that it is essential to assess fear of childbirth in pregnant women as early as possible, up to the 20th week of pregnancy. Early assessment allows for effective identification and intervention, reducing women’s distress and minimizing fear of childbirth before birth^34^.

In the present study, previous pregnancy experiences were associated with fear of childbirth, in line with the results of a meta-synthesis that included 14 qualitative studies and sought to understand the experiences of women with fear of childbirth^28^. The study shows that women suffered consequences from traumatic childbirth experiences, thoughts of previous childbirth, or hearing other women talk about their terrible childbirth experiences. The authors point out that most multiparous women did not feel fear during their first pregnancy, but after experiencing a traumatic childbirth, they were terrified of the experience of another childbirth. They recommend that women who are afraid after a previous negative experience need support to regain confidence in maternity professionals. Primiparous women require similar support to ensure that other women’s experiences will not be repeated with them^35^. It is agreed that health professionals are key messengers who can improve or worsen women’s fear of childbirth, depending on the type of care provided^34^.

Qualitative research conducted in Brazil highlights the myths and fears surrounding childbirth and emphasizes that both pregnant women and new mothers described pain as a factor that could prevent them from choosing natural childbirth. The authors consider that the high importance attributed to the experience of childbirth in women’s lives, which leaves a mark on their lives, justifies the urgent need to review the inhumane practices and interactions that are still present in the posture of so many professionals, replacing them with harmonious interactions^36^.

A systematic review that included 21 studies aimed to identify possible causes and predisposing factors and outcomes of fear of childbirth for women of childbearing age. In the study, the strongest predictive factor for fear of childbirth in multiparous women was a negative experience with normal delivery or cesarean section. Factors such as prolonged labor, use of epidural anesthesia, obstetric complications, presence of traumatic stress symptoms, and need for psychiatric care are outcomes reported in women with high fear of childbirth^16^.

In a literature review conducted by Brazilian researchers^37^, with the objective of reviewing concepts and definitions about the fear of childbirth, the findings were different from those found in our study. The review points out that nulliparous women are more afraid of childbirth than multiparous women, both at the beginning and at the end of pregnancy, and that more advanced gestational age is associated with a higher level of fear of childbirth. The results showed that fear of childbirth is related to an increased risk of adverse obstetric events, such as increased cesarean sections, premature birth, prolonged labor, postpartum depression, and post-traumatic stress. This evidence highlights the importance of discussing fear of childbirth during prenatal care and raises awareness of the need for future strategies to assess and treat this fear^37^.

In our study, being younger, especially under the age of 25.5, was a factor that influenced fear of childbirth. Our findings corroborate different studies. Research conducted in the United States, including 758 young women and students, suggests that women have more knowledge deficits and want to learn more about childbirth. The study also identified that informal channels, such as friends and family, have a major influence on young women’s choices regarding childbirth^14^.

Sociodemographic factors may have little influence on healthcare interventions, but it is essential to consider their impact on people’s conditions when planning childbirth care. Unlike our findings, a systematic review did not identify an association between fear of childbirth and age. The impact of age on fear of childbirth varied across studies: some found no association, while others identified a higher risk in nulliparous women < 32 years and in nulliparous and multiparous women ≥ 40 years. In addition, variation in the size of the populations studied may affect the results^16^.

For this reason, it is recommended that childbirth education be provided well before a young woman’s first birth, when views on maternity care are being established and there is potential to reinforce the notion that a low-intervention birth, followed by a vaginal delivery, is the healthiest and best option for most women of childbearing age^37^.

A cross-sectional study, which aimed to outline the epidemiological profile of fear of childbirth in Brazilian pregnant women, used the Portuguese version of the Wijma Delivery Expectancy/Experience Questionnaire (W-DEQ) and demonstrated a weak positive correlation between the age of the pregnant woman and the score (W-DEQ), suggesting that the older the woman, the greater her levels of fear of childbirth^13^.

Our study evaluated fear in men and women and found that it was associated with the female sex. Although fear of childbirth is often discussed more among women, it is crucial to recognize that the father figure can be a fundamental source of support for the pregnant woman. Many fathers become the main source of emotional and practical support during pregnancy and childbirth.

An integrative review examined and synthesized the results of 17 studies related to paternal fear of childbirth and identified that expectant fathers experience pathological fear regarding childbirth, related to the health and life of the baby, the health and life of their partner, and their own reactions and behaviors during the process. This research revealed that fear of childbirth negatively influences the lives of men and, consequently, their families. The study highlights the need for further research on methods and models for identifying paternal fear, using reliable and culturally validated instruments, targeting men at risk or afraid of childbirth. In addition, it is essential to develop new educational strategies on childbirth, providing appropriate information for this population, as paternal fear of childbirth can influence women’s choice of cesarean section. An intervention that includes the couple may be appropriate in some cases^17^.

A study conducted in the United Kingdom with female students without children to assess the influence of sociocultural representations and perceptions of childbirth revealed that students with negative impressions of childbirth were significantly more likely to have higher scores for fear of childbirth. Negative impressions of pregnancy perceived in friends or family members and perceptions of childbirth represented in visual media were associated with higher scores of fear of childbirth. Students who had experienced witnessing a birth described the experience as incredible, and their scores were lower for fear of childbirth. These findings highlight the potential negative influence of sociocultural representations. They also indicate the need for studies using mixed methods to determine the positive effects of promoting positive images and messages about childbirth and birth within public health initiatives^38^.

Even though the findings of this study point to fear of childbirth predominantly in women, it is understood that interventions for childbirth care also need to be directed at men, since they are also impacted by the components of pregnancy. A study conducted on a sample of Australian men to identify risk factors associated with paternal perinatal mental distress found that sleep disorders, mental distress due to unplanned pregnancy, work-family conflict, stress related to the role of provider, marital distress, and maternal depression are significant risk factors that affect men and increase paternal mental distress during pregnancy. These findings are of great significance for the development of early interventions specific to paternal perinatal care^39^.

In this study, 46.5% of participants were healthcare professionals who expressed fear regarding childbirth. It is important to note that the presence of fear can create obstacles for these professionals when attempting to demystify the negative feelings surrounding the childbirth process. It is essential that these professionals recognize and address their own concerns regarding childbirth to ensure adequate and welcoming care for pregnant women.

Limitations of this study include lower male participation, which may have influenced the gender comparison. In addition, recruitment was conducted in a virtual environment, which may have excluded people in socially vulnerable situations with limited access to the internet, electronic equipment, or low digital proficiency. It should also be noted that the sample was obtained by a non-probabilistic, intentional method, which represents a methodological limitation, as it restricts the possibility of generalizing the findings to the Brazilian population. Although the objective of the study was not inferential, this characteristic should be considered when interpreting the results.

In summary, this study reveals that fear of childbirth is a significant reality among Brazilian adults, reaching a considerable prevalence. The complexity of this phenomenon is evident, with factors such as age and prior pregnancy experience influencing the intensity of fear. Identifying these predictors is crucial, as it provides valuable insights for developing more effective public health strategies. Understanding which groups are most vulnerable to fear of childbirth allows for the creation of targeted interventions aimed at providing psychological and educational support, which can not only improve the childbirth experience but also have a positive impact on reproductive decisions and the mental health of the general population.

## Conclusion

It was observed that fear of childbirth is common among Brazilian adults. Higher scores for fear of childbirth were associated with adults, females, and women who had already been pregnant. The variables that influence fear of childbirth should be considered in health interventions before the onset of pregnancy and during prenatal care.

Understanding the factors associated with fear of childbirth in different populations is essential, as results may vary significantly due to contextual influences. Factors such as social contexts, ethnicity, religion, beliefs, perceptions, and legislation can influence how men and women view childbirth. Each of these elements can affect the expectations and experiences of men and women differently. The importance of culturally sensitive and personalized approaches to prenatal care and emotional support is noteworthy. Analyzing these variables can provide a deeper and more comprehensive understanding, enabling more effective interventions to mitigate fear of childbirth and improve the experience of pregnant women. Based on the findings of this research, it is believed that intervention studies for fear of childbirth should be further developed to create preventive and supportive strategies that promote a more positive experience during pregnancy and childbirth.

## Data Availability

Datasets related to this article will be available upon request to the corresponding author.
