# Supplementary material for: Prevalence and factors associated with fear of childbirth: national online survey of Brazilian adults
Source: Rev Lat Am Enfermagem. 2026 Aug 21;34:e4897. doi: 10.1590/1518-8345.7617.4897 (PMC13505609; doi:10.1590/1518-8345.7617.4897)
Supplement: Translation

# Translation: Prevalência e fatores associados ao medo do parto: inquérito online nacional em adultos brasileiros^*^

**DOI:** 10.1590/1518-8345.7617.4898

**Authors:** Andrezza Belluomini Castro, Rafaela Aparecida Prata, Marla Andréia Garcia de Avila, Milena Temer Jamas

**Affiliations:** 1 Universidade Estadual Paulista, Departamento de Enfermagem, Botucatu, SP, Brasil.

**Keywords:** Enfermagem Obstétrica, Medo, Parto, Internet, Educação em Saúde, Gestante

## Abstract

**Objetivo:**

identificar a prevalência e os fatores associados ao medo do parto em adultos brasileiros.

**Método:**

estudo transversal com 528 participantes entre 18 e 58 anos. Os dados foram coletados por meio de um inquérito online, que abordou características sociodemográficas, comportamentais, além de informações relacionadas à saúde, à história obstétrica e ao medo do parto, conforme a versão brasileira do instrumento *Childbirth Fear Prior to Pregnancy*. As variáveis foram analisadas e ajustadas por modelos de regressão linear múltipla, assumindo que os resíduos seguem uma distribuição normal.

**Resultados:**

a prevalência do medo do parto foi de 28,4% (n=133) para baixo, 19,5% (n=103) para moderado e 26,9% (n=142) para alto medo do parto. Análises multivariadas mostraram que escores mais baixos de medo do parto estavam associados à idade mais jovem (p=0,002) e sexo feminino (p<0,000). Por outro lado, indivíduos com gestação anterior tiveram maiores escores do CPFF (p=0,005).

**Conclusão:**

a prevalência do medo do parto foi 71,6% (n=378), distribuída entre baixo (25,2%), moderado (19,5%) e alto medo (26,9%). Fatores como maior idade e gênero feminino foram associados a menores escores de medo, enquanto a gestação prévia esteve ligada a um nível mais elevado de medo. Esses resultados sugerem que a experiência materna pode influenciar decisões futuras sobre a escolha do próprio parto ou impactar a percepção de outras pessoas do seu convívio.

## Destaques

**(1)** Escores mais altos de medo do parto mostraram-se associados ao sexo feminino. **(2)** Prevalência de medo do parto na população que deseja ter filhos. **(3)** A população mais jovem apresenta mais medo do parto. **(4)** Maior associação ao medo do parto em mulheres com experiência anterior.

## Introdução

O medo do parto é caracterizado por sentimento de insegurança e ansiedade antes, durante ou após o nascimento, em relação ao processo de trabalho de parto e ao momento de dar à luz, com variações de níveis mais leves ou mais graves^1^ ^-^ ^2^. Esse sentimento pode ser decorrente de relatos ou experiências ruins em partos anteriores, geradas por tratamento hostil pela equipe, sensação de abandono ou não participação das decisões em relação à assistência^3^ ^-^ ^4^.

O medo intenso do parto é uma questão multifacetada que se estende muito além do evento obstétrico em si, impactando significativamente a saúde mental e o bem-estar emocional das mulheres a longo prazo. Conforme evidenciado pela literatura, a complexidade do parto, que mobiliza uma gama de sentimentos contraditórios como ansiedade, insegurança e estresse, já aponta para a relevância dos aspectos psicológicos envolvidos^5^. 

As evidências científicas indicam que fatores socioculturais desempenham um papel significativo no desencadeamento do medo do parto. Narrativas culturais, transmitidas por familiares e amigos, que associam o parto vaginal à dor e ao sofrimento, contribuem para que as mulheres desenvolvam ansiedade, insegurança e temores intensificados. Isso resulta na subestimação da própria capacidade fisiológica de dar à luz, apesar da história milenar de partos naturais sem medicalização. O grande desafio na assistência obstétrica é, portanto, devolver à gestante o controle sobre seu corpo e pensamentos, promovendo essa confiança ao longo de todo o pré-natal, e não apenas nas horas que antecedem o parto^6^. 

Essa apreensão profunda pode gerar uma série de consequências que se manifestam de diversas formas na vida das mulheres. Além dos efeitos fisiológicos já conhecidos - como prolongamento da dilatação, maior risco de pré-eclâmpsia, parto prematuro e cesariana de emergência -, o medo do parto está intrinsecamente ligado a resultados negativos de saúde mental e qualidade de vida. Ele pode levar ao desenvolvimento de depressão pós-parto, afetar os índices de amamentação e até mesmo resultar em maior necessidade de admissão do recém-nascido em unidades de terapia intensiva^7^ ^-^ ^8^.

Esse estudo identificou que a primeira gestação, menor nível educacional, concepção natural e preferência por cesariana estão associados a um maior nível de medo. Além disso, comorbidades como alta miopia e doenças urinárias foram apontadas como preditores independentes de medo severo. Reconhecer a complexidade do medo do parto é crucial para os profissionais de saúde que integram a triagem obstétrica nos cuidados pré-natais, especialmente em grupos de risco. Isso permite oferecer aconselhamento e apoio personalizados, promovendo escolhas de parto informadas e contribuindo para o bem-estar psicológico materno e melhores resultados obstétricos^9^. 

A Suécia é um dos poucos países em que o medo do parto é reconhecido como um problema importante e onde serviços de aconselhamento estão disponíveis para a maioria das mulheres grávidas. Uma pesquisa realizada em 2006, com 2.262 mulheres suecas, indicou a prevalência do medo do parto em torno de 10%. As mulheres eram recrutadas em sua primeira consulta a uma clínica pré-natal durante três semanas predeterminadas, distribuídas ao longo de 1 ano. O estudo revelou que o medo do parto estava associado a um aumento da taxa de cesarianas eletivas e com experiência de parto “aceitável” nas mulheres que realizaram aconselhamento^4^.

Em um estudo realizado na Croácia, envolvendo estudantes universitárias jovens e não grávidas dos cursos de ciências da saúde, ciências sociais e humanas, demonstrou-se que 25,9% das estudantes relataram medo significativo do parto, sendo que o das estudantes de ciências da saúde era em nível menor quando comparado com o das demais estudantes dos outros programas, além de terem recebido mais informações sobre parto no contexto familiar quando comparado ao processo de aprendizagem. Acredita-se que o nível mais alto de medo do parto poderia ser previsto na identificação de fatores como a sensibilidade à ansiedade, traço de ansiedade, expectativa de dor no parto e fontes de conhecimento sobre parto^10^.

O medo do parto afeta negativamente as mulheres de várias maneiras, o que pode desencadear o aumento do uso de analgesia de parto, desequilíbrio emocional, prolongamento do trabalho de parto, a escolha por uma cesárea eletiva, dificuldade de vínculo com o bebê e depressão pós-parto^11^ ^-^ ^14^. Adicionalmente, isso pode impactar a harmonia nas relações familiares e com o parceiro. Há evidências substanciais indicando que altos níveis de medo do parto acarretam transtorno de estresse pós-traumático, ansiedade pós-parto, depressão e psicose, e até mesmo aumentam a necessidade de cuidados psiquiátricos a longo prazo^15^ ^-^ ^16^.

Evidências crescentes sugerem que o medo do parto não é uma prerrogativa exclusiva das mulheres; os futuros pais também podem vivenciar ansiedades significativas relacionadas ao nascimento, embora suas preocupações e manifestações possam diferir. A inclusão da perspectiva masculina na investigação do medo do parto é fundamental, não apenas para compreender a experiência paterna em si, mas também devido ao impacto que o bem-estar do pai pode ter sobre a parceira, a dinâmica familiar e o próprio processo de transição para a parentalidade^17^. Ignorar o medo do parto nos homens significa deixar uma parcela da população sem o suporte e a compreensão necessários durante um período de vulnerabilidade.

A adoção de medidas de avaliação e intervenção sobre o parto contribui para a desmistificação de medos e crenças errôneas, melhorando o bem-estar e a qualidade de vida dos jovens, além de reforçar os esforços internacionais na redução das taxas de cesarianas eletivas e da medicalização. É importante avaliar e intervir previamente em populações não grávidas, uma vez que é no período gestacional que os medos se tornam mais intensos^18^.

Nesse cenário, a enfermagem assume um papel central e insubstituível. Profissionais de enfermagem, com sua proximidade e abordagem holística no cuidado perinatal, estão em uma posição privilegiada para atuar como facilitadores no processo de desmistificação do parto e na redução do medo associado. Por meio de uma escuta ativa, educação perinatal abrangente, e implementação de práticas de cuidado personalizadas e fundamentadas em evidências científicas, a enfermagem pode empoderar tanto mulheres quanto homens. Isso envolve desconstruir mitos, fornecer informações claras e realistas sobre o processo de nascimento, discutir opções de manejo da dor, promover o protagonismo do casal e criar um ambiente de confiança e segurança^19^ ^-^ ^21^.

Uma pesquisa qualitativa^22^ investigou a percepção de mulheres sobre o aconselhamento de parteiras em relação ao medo do parto. Os resultados revelaram uma melhora na confiança das mulheres para o parto após a aquisição de informações e conhecimentos. As participantes relataram sentir maior calma e preparação, o que fortaleceu sua capacidade de lidar com a incerteza do processo de parturição, impactando positivamente a experiência de dar à luz. Adicionalmente, o sentimento de segurança proporcionado pelo apoio profissional durante o parto contribuiu para que as mulheres se sentissem fortalecidas. Essa experiência positiva, por sua vez, reforçou a autoconfiança para partos futuros, e o medo do parto foi percebido como reduzido ou controlável. 

Apesar de sua comprovada eficácia na redução da dor, diminuição do tempo de trabalho de parto e do parto em si, e por serem de baixo custo e fácil aplicabilidade, métodos não farmacológicos como terapia térmica, massagem, aromaterapia, acupressão, dança e exercícios com bola suíça ainda são pouco divulgados. Essas abordagens também estão associadas a um menor uso de intervenções medicamentosas para analgesia. No entanto, é no cenário do parto, com a presença e atuação da enfermagem obstétrica, que esses métodos ganham maior visibilidade e são mais amplamente empregados^23^ ^-^ ^24^. A enfermagem desempenha um papel crucial como facilitadora na desmistificação do parto e na redução do medo, promovendo ativamente essas práticas de cuidado personalizadas e baseadas em evidências, que empoderam a mulher e contribuem para uma experiência mais positiva e menos medicalizada do parto. 

Torna-se imperativo investigar os fatores associados ao medo do parto, ampliando o escopo tradicional para incluir a população masculina e considerando a avaliação em fases que antecedem a gestação, particularmente no contexto brasileiro. A compreensão aprofundada desses fatores é fundamental para subsidiar o desenvolvimento de estratégias de intervenção mais eficazes, inclusivas e culturalmente sensíveis, promovidas especialmente pela enfermagem, visando aprimorar a saúde perinatal e a experiência de nascimento para todos os envolvidos. Desse modo, esse estudo teve como objetivo identificar a prevalência e os fatores associados ao medo do parto em adultos brasileiros.

## Método

### Delineamento do estudo

Trata-se de um estudo transversal e analítico, realizado com um inquérito online com adultos acima de 18 anos, brasileiros das cinco macrorregiões do país, norteado pela diretriz *Strengthening the Reporting of Observational Studies in Epidemiology* (STROBE)^25^, realizado no período de janeiro a abril de 2022 por meio do formulário digital disponibilizado pela ferramenta Google *Forms*.

### Definição da amostra 

O estudo incluiu homens e mulheres acima de 18 anos, independentemente de seu estado conjugal, com intenção de ter filhos no futuro. Foram excluídos os participantes que não responderam ao instrumento no período da coleta de dados e aqueles cujos formulários não tiveram todas as perguntas respondidas. 

Diante da população-alvo, formada por adultos acima de 18 anos que tinham intenção de ter filhos no futuro, residindo em qualquer região do país, a amostra é classificada como não probabilística do tipo intencional. É prudente considerar que o plano amostral adotado de amostragem não probabilística tipo intencional de 528 participantes pode gerar erros sistemáticos de seleção que influenciam nas estimativas das associações entre os fatores investigados e o desfecho do escore do medo do parto, sobretudo se considerarmos que a intenção é a inferência à população brasileira.

Contudo, para a estimativa do seu tamanho, considerou-se que a intenção era conhecer um processo e não inferir para uma população especificamente delimitada no tempo-espaço. Sendo assim, para o cálculo do número mínimo de participantes (e não para o tamanho mínimo de uma amostra representativa de uma população-alvo), assumiu-se prevalência de 0,50, nível de 5% de significância e precisão de 3%. 

### Variáveis do estudo

As variáveis independentes foram: dados sociodemográficos como idade (em anos); gênero (masculino; feminino; prefiro não identificar); cor da pele autorreferida (branco/a; preto/a; pardo/a; indígena; amarelo/a); escolaridade (sem escolaridade; ensino fundamental incompleto; ensino fundamental completo; ensino médio incompleto; ensino médio completo; ensino superior; pós-graduação); procedência (norte; nordeste; sul; sudeste; centro-oeste); profissional da saúde (sim ou não); situação conjugal (com companheiro/a ou sem companheiro/a). As variáveis obstétricas: possui filhos (sim ou não); tipo de parto da última gestação (normal; cesariana); vivenciou experiência negativa na gestação anterior (sim; não); variáveis comportamentais: experiências de amigos ou familiares influenciaram o tipo de parto que escolheu (sim; não); fonte de informação da população estudada: onde busca informações sobre parto (vídeos/internet; material impresso; parentes e amigos; profissional de saúde; artigos científicos; não busco informações sobre parto). Neste estudo, considerou-se como variável dependente a escala *Childbirth Fear Prior to Pregnancy* (CFPP) como instrumento de avaliação do medo do parto por permitir a avaliação do medo antes da concepção, em homens e mulheres, sendo reconhecido por sua linguagem acessível, de fácil aplicação, auxiliando o desenvolvimento de intervenções direcionadas. Suas dimensões abordam o medo da dor do parto, o medo das complicações relacionadas ao bebê e o medo de danos corporais^18^.

### Coleta de dados

Para este estudo, os pesquisadores desenvolveram um instrumento de coleta de dados virtual utilizando a plataforma Google *Forms*. Esse questionário foi composto por 26 questões fechadas para levantar informações sobre características sociodemográficas, obstétricas e comportamentais dos participantes (variáveis independentes). 

A versão brasileira online da CFPP foi adotada como instrumento de medida da variável dependente, por ser o único instrumento validado para a população brasileira na avaliação do medo do parto em homens e mulheres^26^. Outro ponto relevante são suas características psicométricas adequadas, incluindo uma linguagem clara e de fácil compreensão, o que facilita sua aplicação. Essas qualidades tornam a CFPP uma ferramenta valiosa não apenas para a pesquisa, mas também para o desenvolvimento de intervenções eficazes e direcionadas à redução do medo do parto^18^.

A versão online brasileira da CFPP é um instrumento de medida curta e simples, unidimensional, de 10 itens, com 5 opções de respostas que variam entre (1) discordo totalmente, (2) discordo, (3) discordo parcialmente, (4) concordo e (5) concordo totalmente, dispostas na vertical. A pontuação final é obtida pela soma da pontuação dos 10 itens, sendo a pontuação mínima 10 e a máxima 50^26^.

A coleta de dados foi realizada por meio de um inquérito online, seguindo as recomendações da lista CHERRIES (*Checklist for Reporting Results of Internet E-Surveys*)^27^, que possibilitou, em um momento que exigia a necessidade de distanciamento social, o alcance de uma amostra diversificada por meio da internet, com abrangência de indivíduos em diferentes regiões geográficas do Brasil e de forma imediata^28^.

A coleta de dados foi realizada pelos pesquisadores e três alunos de graduação, colaboradores da pesquisa. Os participantes foram recrutados por meio da divulgação do formulário digital, disponibilizado via Google *Forms*, em diversos canais online, incluindo e-mails, redes sociais (Facebook e Instagram) e contatos pessoais via WhatsApp. Esse processo expandiu-se por meio de um efeito multiplicador, denominado “bola de neve” ou amostragem de rastreamento de links. A amostragem em bola de neve é uma técnica não probabilística que gera um efeito multiplicador na coleta de dados, similar ao crescimento de uma bola de neve. O processo começa com a identificação de um ou mais “informantes-chave”. Estes, por sua vez, indicam novos participantes de suas redes de contato que se enquadram nos critérios da pesquisa, criando uma cadeia de referências que se expande exponencialmente. Essa abordagem é particularmente eficaz para acessar grupos de difícil alcance ou que abordam temas sensíveis, pois aproveita as conexões existentes entre os indivíduos^29^.

### Análise dos dados

Os dados coletados foram transportados e organizados no programa Microsoft Office Excel. As análises estatísticas foram realizadas no *software* IBM - *Statistical Package for the Social Sciences* (SPSS versão 21).

Primeiramente, realizou-se análise exploratória dos dados com a finalidade de identificar e tratar valores atípicos e identificar o mecanismo de geração de *missings*. Após avaliação inicial, realizou-se análise bivariada para investigar a associação isolada de cada variável independente com a pontuação da escala CFPP por meio de regressões lineares simples com resposta com distribuição probabilística normal (que acomoda desfechos numéricos com grau de simetria no mínimo razoável).

O corte p < 0,20 é utilizado para evitar deixar de fora uma variável que possua um papel importante e consolidado dentro de um modelo de determinação social ou dentro da patogênese do desfecho somente por causa de uma associação mais fraca. Em outras palavras, prioriza os paradigmas da saúde ao invés de um critério estatístico. No modelo de regressão múltipla, as associações foram consideradas estatisticamente significativas se p < 0,05. 

### Aspectos éticos

Todas as atividades foram desenvolvidas após a aprovação do Comitê de Ética em Pesquisa da Faculdade de Medicina de Botucatu, sob o parecer n.º 4.836.471, em conformidade com a Resolução do Conselho Nacional de Saúde n.º 510/2016. A aceitação em participar da pesquisa foi registrada por meio do Termo de Consentimento Livre e Esclarecido (TCLE) no formato digital^30^. 

## Resultados

Aceitaram participar do estudo 671 indivíduos. No entanto, 143 participantes foram excluídos por não atenderem aos critérios de inclusão, tendo o estudo como amostra final 528 participantes das cinco macrorregiões do Brasil.

Dos participantes, 81,6% (n=431) são do sexo feminino, com mediana de 24 anos (mínima de 18 e máxima de 58 anos), 69,3% (n=366) se autodeclararam brancos ou amarelos, 75,8% (n=400) com no mínimo o ensino médio, 50,9% (n=269) eram profissionais da saúde, 61% (n=322) referiram ter companheiro(a). A região Sudeste foi a mais representada, com 62,1% (n=328) dos participantes, conforme a Tabela 1.

**Tabela 1:** Caracterização sociodemográfica dos participantes (n = 528). Brasil, 2024

Variáveis	Total	
	n*	%^†^
**Gênero**		
Feminino	431	81,6
Masculino	96	18,2
Não identificou	1	0,2
**Raça/Cor**		
Branca/Amarela	366	69,3
Preta/Parda/Indígena	162	30,7
**Escolaridade**		
Ensino fundamental e médio incompletos	128	24,2
Ensino médio, Graduação e Pós-graduação	400	75,8
**Situação conjugal**		
Com companheiro	322	61,0
Sem companheiro	206	39,0
**Região**		
Sudeste	328	62,1
Centro-Oeste	83	15,7
Nordeste	57	10,8
Sul	35	6,6
Norte	25	4,7
**Profissional da saúde**		
Sim	269	50,9
Não	259	49,1

*n = Número de participantes; ^†^% = Porcentagem

Dos 95 participantes que referiram ter filhos, 29,4% dos participantes (n=28) tiveram experiência com o parto normal ou vivenciaram com seus parceiros. Além disso, 33 (6,3%) dos participantes relataram experiências negativas na última gestação e 71,6% (n=378) referiram medo do parto. A busca de informações sobre o parto foi referida por 74,6% (n=394), sendo que 26,3% (n=139) buscam essas informações principalmente com profissionais de saúde e 22,2% (n=117) na internet e em vídeos, conforme a Tabela 2.

**Tabela 2:** Características obstétricas e percepções sobre parto e fonte de informações entre adultos. Brasil, 2024

Variáveis	n*	%^†^
Tem filhos (n=528)	95	18,0
Parto vaginal na última gestação (Sim) (n=95)	28	29,5
Experiência negativa na última gestação (Sim) (n=95)	33	34,7
Experiências de amigos e família influenciam a escolha do tipo de parto (Sim) (n=528)	123	23,3
Medo do parto (Sim) (n=528)	378	71,6
Busca informações sobre parto (Sim) (n=528)	394	74,6
**Locais em que buscam as informações**		
Profissional da saúde	139	26,3
Vídeos/internet	117	22,2
Artigos científicos	79	15,0
Parentes e amigos	54	10,2
Material impresso	05	0,9

*n = Número de participantes; ^†^% = Porcentagem

A Tabela 3 mostra os escores dos participantes para o medo do parto, seguindo a classificação da CFPP. Verificou-se que a prevalência do medo do parto foi de 71,6% (n=378), sendo 25,2% (n=133) baixo, 19,5% (n=103) moderado e 26,9% (n=142) alto medo do parto.

**Tabela 3:** Classificação do medo do parto em adultos segundo CFPP* (n = 528). Brasil, 2024

Classificação	Pontuação CFPP*	n^†^	%^‡^
Ausência de medo	10 ≤ x ≤ 28	150	28,4
Baixo medo	29 ≤ x ≤ 33	133	25,2
Medo moderado	34 ≤ x ≤ 37	103	19,5
Alto medo	38 ≤ x ≤ 50	142	26,9

*Versão da escala *Childbirth Fear Prior to Pregnancy* com pontuação de 10 a 50; ^†^n = Número de participantes; ^‡^% = Porcentagem

Conforme os resultados apresentados na Tabela 4, a análise bivariada investigou a associação entre variáveis independentes e o escore da escala CFPP. Apresentaram associações estatisticamente significativas (p < 0,20) as variáveis idade (p < 0,001), sexo feminino (p < 0,001), não ser profissional da saúde (p = 0,027), residir nas regiões Sudeste (p=0,130) e Nordeste (p=0,150), situação conjugal com companheiro (p=0,137), não ter filhos (0,051), ter mais de uma gestação (p=0,001), experiência de amigos/família na escolha do tipo de parto (p=0,017), buscar informações em artigos científicos (p= 0,035), parentes e amigos (p=0,027), profissionais de saúde (p=0,007) e vídeos/internet (p=0,007).

**Tabela 4:** Associação bivariada entre variáveis sociodemográficas, obstétricas, comportamentais e o escore da CFPP* (n = 528). Brasil, 2024

Variáveis	b^†^	IC^‡^95%		*p* ^*§*^
		Limite Inferior	Limite Superior	
Idade	-0,17	-,25	-,08	**0,000**
Gênero				
Masculino	3,80	-10,95	18,56	0,614
Feminino	-3,48	-5,14	-1,81	**0,000**
Outra classificação^||^				
Raça ou Cor				
Branco/Amarelo	0,32	-1,09	1,74	0,654
Preta/Parda/Indígena^¶^				
Região
Centro-Oeste	-2,35	-6,23	1,54	0,236
Sudeste	2,46	-,72	5,65	**0,130**
Sul	1,18	-1,81	4,17	0,441
Nordeste	1,94	-,70	4,58	**0,150**
Norte**				
Não ser profissional da saúde	-1,47	-2,77	-0,17	**0,027**
Trabalho de parto	-1,89	-4,85	1,06	0,210
Escolaridade mínima ensino médio	0,23	-1,29	1,75	0,768
Situação conjugal com companheiro	-1,01	-2,35	0,32	**0,137**
Não ter filhos	-1,69	-3,38	0,00	**0,051**
Ter mais de uma gestação	4,94	2,06	7,82	**0,001**
Experiência negativa na última gestação	-0,22	-2,91	2,47	0,871
Experiência de amigos/família na escolha do tipo de parto	1,08	-,46	2,62	**0,170**
Buscar informações sobre o parto	-0,30	-1,80	1,19	0,691
Locais que buscam informações				
Artigos científicos	-1,95	-3,77	-0,13	**0,035**
Materiais impressos	-1,39	-8,11	5,34	0,687
Parentes, amigos	2,42	,27	4,56	**0,027**
Profissionais de Saúde	-2,01	-3,48	-,54	**0,007**
Vídeo/Internet	2,15	,59	3,71	**0,007**

*Versão da escala *Childbirth Fear Prior to Pregnancy;* ^*†*^Intercepto; ^‡^Intervalo de confiança; ^§^Análise bivariada por regressão linear simples; ^||^Referência para gênero; ^¶^Referência para cor/raça; **Referência para regiões no Brasil

Foi realizada uma análise multivariada por regressão linear múltipla, com o escore total da escala CFPP como variável dependente (pontuação variando de 10 a 50). Os resultados demonstraram que, quanto maior for a idade, menor a pontuação obtida na escala, ou seja, menor o medo do parto (b = -0,17; IC95% -0,25 a -0,08; p < 0,001). Além disso, os homens apresentaram pontuação em média 3,6 pontos menor, quando comparados com as mulheres, demonstrando menor medo do parto (b = -3,48; IC95% -5,14 a -1,81; p < 0,001). Por outro lado, participantes com experiência gestacional anterior maiores escores foram associados a participantes que relataram experiência de parto; apresentaram em média 4,5 pontos a mais, evidenciando um maior medo do parto (b = 4,52; IC95% 1,35 a 7,68; p = 0,005) (Tabela 5).

**Tabela 5:** Regressão linear múltipla entre variáveis sociodemográficas, obstétricas, comportamentais e o escore total da escala CFPP* (n = 528). Brasil, 2024

Variáveis	b^†^	IC^‡^95%		*p* ^*§*^
		Limite Inferior	Limite Superior	
Idade	-0,16	-,26	-,06	**0,002**
Gênero				
Masculino	1,87	-12,22	15,96	0,795
Feminino	-3,68	-5,32	-2,04	**0,000**
Outra classificação^||^				
Região
Centro-Oeste	-1,89	-5,65	1,87	0,324
Sudeste	0,97	-2,09	4,03	0,534
Sul	0,77	-2,09	3,64	0,597
Nordeste	1,17	-1,37	3,71	0,365
Norte^¶^				
Não ser profissional da saúde	-1,22	-2,54	0,11	0,072
Situação conjugal com companheiro	0,13	-1,25	1,50	0,855
Não ter filhos	1,26	-,98	3,50	0,271
Ter mais de uma gestação	4,52	1,35	7,68	**0,005**
Experiência de amigos/família na escolha do tipo de parto	1,45	-,07	2,97	0,062
Buscam informações com parentes, amigos	1,58	-0,76	3,93	0,186
Buscam informações em artigos científicos	1,60	-3,70	,50	0,134
Buscam informações com profissionais de saúde	-1,43	-3,18	,33	0,110
Buscam informações com vídeo/internet	0,82	-,99	2,62	0,376

*Versão da escala *Childbirth Fear Prior to Pregnancy;* ^*†*^Intercepto; ^‡^Intervalo de confiança; ^§^Análise bivariada por regressão linear múltipla; ^||^Referência para gênero; ^¶^Referência para regiões no Brasil

## Discussão

O destaque do presente estudo foi buscar evidências, por meio de um instrumento apropriado ao contexto brasileiro, que pudessem contribuir para a identificação de fatores associados ao medo do parto normal na população brasileira, incluindo homens e mulheres, fornecendo subsídios para a realização de materiais educativos e ações de políticas na temática.

A tocofobia, ou medo intenso do parto, afeta aproximadamente 14% das mulheres globalmente^11^. Ao analisar a prevalência em diferentes países, observa-se uma variação significativa. Nos Estados Unidos, por exemplo, a condição é mais incidente, afetando 26,9% das gestantes^31^. Já em nações como Austrália e Finlândia, a taxa de medo do parto situa-se entre 20% e 25%^32^ ^-^ ^33^. Esses dados ressaltam a importância de se compreender e abordar a tocofobia, considerando suas particularidades culturais e geográficas.

Um estudo qualitativo realizado no Reino Unido, incluindo 10 gestantes e 13 parteiras, identificou elementos importantes que influenciam o medo do parto, tais como o medo de não conseguir lidar com o imprevisível; medo de que o bebê ou a própria gestante se machuque no parto; medo de não conseguir dar à luz; medo de não saber lidar com a dor; medo de não ter autonomia para tomada de decisão; medo de intervenções; medo do abandono e medo de perder o controle. Nesse estudo, ao serem questionadas sobre quando deveriam ser feitas perguntas sobre o medo do parto, a maioria respondeu que o mais precocemente possível. Destacou que é essencial avaliar o medo do parto em gestantes o mais precocemente possível, indicando até a 20ª semana de gestação. A avaliação precoce permite identificar e intervir de maneira eficaz, reduzindo a angústia das mulheres e minimizando o medo do parto antes do nascimento^34^. 

No presente estudo, experiências prévias de gestação mostraram associação ao medo do parto, indo ao encontro dos resultados de metassíntese que incluiu 14 estudos qualitativos e buscou compreender as vivências de mulheres com medo do parto^28^. O estudo mostra que as mulheres sofreram consequências de experiências traumáticas de parto, pensamentos de parto anterior ou de ouvir outras mulheres contarem sobre sua terrível experiência de parto. Os autores destacam que a maioria das multíparas não sentiu medo durante a primeira gravidez, mas, depois de terem vivenciado um parto traumático, ficaram apavoradas com a experiência de outro parto. Recomendam que mulheres com medo após uma experiência negativa anterior precisam de apoio para poder recuperar a confiança nos profissionais da maternidade. Primigestas exigem apoio semelhante para garantir que as experiências de outras mulheres não serão repetidas com elas^35^. Concorda-se que os profissionais de saúde são mensageiros-chave que podem melhorar ou piorar o medo do parto em mulheres, a depender do modo de cuidado realizado^34^.

Investigação qualitativa conduzida no Brasil sinaliza os mitos e medos que permeiam o nascimento e destaca que tanto gestantes como puérperas verbalizaram a dor como fator que poderia impedir a preferência pelo parto normal. Os autores consideram que o alto grau atribuído à experiência do nascimento na vida das mulheres, que deixa marcas em suas vidas, é justificativa para a urgente necessidade de revisar as práticas e interações desumanas que ainda estão presentes na postura de tantos profissionais, substituindo-as por interações harmoniosas^36^.

Revisão sistemática que incluiu 21 estudos objetivou identificar possíveis causas e fatores predisponentes e desfechos do medo do parto para mulheres em idade fértil. No estudo, o fator preditivo mais forte para medo do parto em mulheres multíparas foi a experiência negativa no parto normal ou cesárea. Fatores como trabalho de parto prolongado, uso de peridural, complicações obstétricas, presença de sintomas de estresse traumático e necessidade de cuidados psiquiátricos são resultados relatados em mulheres com alto medo do parto^16^.

Em revisão de literatura conduzida por pesquisadores brasileiros^37^, com o objetivo de revisar conceitos e definições sobre o medo do parto, os achados foram diferentes dos encontrados em nosso estudo. A revisão aponta que mulheres nulíparas têm mais medo do parto do que as multíparas, tanto no início quanto no final da gravidez, e que a idade gestacional mais avançada está associada a um maior nível de medo do parto. Os resultados demonstraram que o medo do parto está relacionado a um risco aumentado de eventos obstétricos adversos, como aumento de cesáreas, parto prematuro, trabalho de parto prolongado, depressão pós-parto e estresse pós-traumático. Essas evidências destacam a importância da discussão sobre o medo do parto no pré-natal e acendem um alerta para a necessidade de estratégias futuras para a avaliação e tratamento desse medo^37^. 

Em nosso estudo, ser mais jovem, sobretudo com menos de 25,5 anos, foi um fator que influenciou o medo do parto. Nossos achados corroboram diferentes pesquisas. Investigação conduzida nos Estados Unidos da América, incluindo 758 mulheres jovens e estudantes, sugere que as mulheres apresentam mais déficits de conhecimento e desejam aprender mais sobre o parto. O estudo também identificou que os canais informais, como amigos e familiares, exercem grande influência nas escolhas de mulheres jovens em relação ao parto^14^.

Os fatores sociodemográficos podem ter pouca influência nas intervenções de cuidados de saúde, mas é essencial considerar sua influência nas condições das pessoas ao planejar cuidados para o parto. Diferentemente dos nossos achados, revisão sistemática não identificou associação entre medo do parto e idade. O impacto da idade no medo do parto variou entre os estudos: alguns não encontraram associação, enquanto outros identificaram maior risco em mulheres nulíparas < 32 anos e em mulheres nulíparas e multíparas ≥ 40 anos. Além disso, a variação no tamanho das populações estudadas pode ter implicações nos resultados^16^.

Por essa razão, é recomendado que a educação para o parto seja realizada bem antes do primeiro parto de uma mulher jovem, quando as visões sobre a assistência à maternidade estão sendo estabelecidas e há potencial para reforçar a noção de que um parto de baixa intervenção, seguido de um parto vaginal, é a opção mais saudável e melhor para a maioria das mulheres em idade fértil^37^. 

Um estudo transversal, que objetivou traçar o perfil epidemiológico do medo do parto em gestantes brasileiras, utilizou a versão portuguesa do *Wijma Delivery Expectancy/Experience Questionnaire* (W-DEQ) e demonstrou fraca correlação positiva entre a idade da gestante e a pontuação (W-DEQ), sugerindo que, quanto mais velha for a mulher, maiores os seus níveis de medo do parto^13^.

Nosso estudo avaliou o medo de homens e mulheres, sendo o medo associado ao sexo feminino. Embora o medo do parto seja frequentemente mais discutido entre as mulheres, é crucial reconhecer que a figura paterna pode ser um suporte fundamental para a gestante. Muitos pais se tornam o principal apoio emocional e prático durante a gestação e o parto.

Uma revisão integrativa examinou e sintetizou os resultados de 17 pesquisas relacionadas ao medo do parto paterno e identificou que os futuros pais experimentam um medo patológico em relação ao parto, relacionado à saúde e vida do bebê, à saúde e vida da parceira e às suas próprias reações e comportamentos durante o processo. Esta investigação revelou que o medo do parto influencia negativamente a vida dos homens e, consequentemente, a de suas famílias. O estudo destaca a necessidade de uma investigação mais aprofundada sobre métodos e modelos de identificação do medo paterno, com o uso de instrumentos confiáveis e validados para a cultura local, direcionados a homens em risco ou com medo do parto. Além disso, é essencial desenvolver novas estratégias educacionais sobre o parto, fornecendo informações apropriadas para essa população, pois o medo paterno do parto pode influenciar a escolha das mulheres para cesariana. Uma intervenção que inclua o casal pode ser apropriada em alguns casos^17^. 

Um estudo realizado no Reino Unido com estudantes do sexo feminino e sem filhos para avaliar a influência das representações socioculturais e as percepções sobre o parto revelou que estudantes com impressões negativas sobre o nascimento eram significativamente mais propensas a apresentar escores mais elevados de medo do parto. As impressões negativas de gravidez percebidas em amigos ou familiares e as percepções de nascimento representadas na mídia visual foram associadas a escores mais altos de medo do parto. As estudantes que tiveram experiência de assistir a um nascimento descreveram a experiência como incrível, e suas pontuações eram mais baixas para o medo do parto. Esses achados destacam a potencial influência negativa das representações socioculturais. Eles também indicam a necessidade de estudos com métodos mistos para determinar os efeitos positivos da promoção de imagens e mensagens positivas de parto e nascimento dentro de iniciativas de saúde pública^38^. 

Mesmo com os achados desse estudo apontando o medo do parto predominantemente no sexo feminino, entende-se que as intervenções para assistência ao parto também precisam ser direcionadas aos homens, uma vez que eles também são impactados pelos componentes da gestação. Um estudo realizado em uma amostra de homens australianos para identificar os fatores de risco associados ao sofrimento mental perinatal paterno verificou que distúrbios de sono, sofrimento mental devido à gravidez não planejada, conflito trabalho-família, estresse relacionado ao papel de provedor, sofrimento conjugal e depressão materna são fatores de risco significativos que afetam os homens e aumentam o sofrimento mental paterno durante a gravidez. Estes achados são de grande significado para o desenvolvimento de intervenções precoces específicas à assistência perinatal paterna^39^. 

Neste estudo, 46,5% dos participantes eram profissionais da saúde, que demonstraram medo em relação ao parto. É importante destacar que a presença do medo pode criar obstáculos para esses profissionais ao tentarem desmistificar os sentimentos negativos que cercam o processo de parto. É essencial que esses profissionais reconheçam e abordem suas próprias preocupações em relação ao parto, a fim de garantir uma assistência adequada e acolhedora às gestantes.

Como limitações desta pesquisa, destaca-se a menor participação de indivíduos do sexo masculino, o que pode ter influenciado a análise comparativa entre os gêneros. Além disso, o recrutamento foi realizado em ambiente virtual, o que pode ter excluído pessoas em situação de vulnerabilidade social, com acesso limitado à internet, equipamentos eletrônicos ou com baixa proficiência digital. Também se ressalta que a amostra foi obtida por método não probabilístico do tipo intencional, o que representa uma limitação metodológica, pois restringe a possibilidade de generalização dos achados para a população brasileira como um todo. Ainda que o objetivo do estudo não tenha sido inferencial, essa característica deve ser considerada na interpretação dos resultados.

Em síntese, este estudo revela que o medo do parto é uma realidade expressiva entre adultos brasileiros, atingindo uma prevalência considerável. Destaca-se a complexidade desse fenômeno, com a influência de fatores como idade e experiência gestacional prévia na intensidade do medo. A identificação desses preditores é crucial, pois oferece subsídios valiosos para o desenvolvimento de estratégias de saúde pública mais eficazes. Compreender quem são os grupos mais vulneráveis ao medo do parto permite que intervenções direcionadas sejam criadas, visando oferecer suporte psicológico e educacional, o que pode não só melhorar a experiência do parto, mas também ter um impacto positivo nas decisões reprodutivas e na saúde mental da população em geral.

## Conclusão

Observou-se que o medo do parto é frequente entre adultos brasileiros. Os escores mais altos do medo do parto mostraram-se estar associados a adultos, ao sexo feminino e a mulheres que já haviam tido uma gestação. As variáveis que exercem influência no medo do parto devem ser valorizadas nas intervenções de saúde, antes do início da gestação e durante o pré-natal.

Compreender os fatores associados ao medo do parto em diferentes populações é essencial, pois os resultados podem variar significativamente devido a influências contextuais. Fatores como contextos sociais, etnias, religiões, crenças, percepções e legislações podem influenciar o olhar de homens e mulheres para o parto. Cada um desses elementos pode afetar as expectativas e experiências de homens e mulheres de maneiras distintas. Destaca-se a importância de abordagens culturalmente sensíveis e personalizadas no cuidado pré-natal e no apoio emocional. Analisar essas variáveis pode proporcionar uma compreensão mais profunda e abrangente, permitindo intervenções mais eficazes para mitigar o medo do parto e melhorar a experiência das gestantes. Com base nos achados desta pesquisa, acredita-se que estudos de intervenção para o medo do parto devem ser aprofundados para o desenvolvimento de estratégias preventivas e de suporte que promovam uma experiência mais positiva durante a gestação e o parto.

## Data Availability

Os conjuntos de dados relacionados a este artigo estarão disponíveis mediante solicitação ao autor correspondente.

# Translation: Prevalencia y factores asociados al miedo al parto: una encuesta nacional en línea a adultos brasileños^*^

**DOI:** 10.1590/1518-8345.7617.4896

**Authors:** Andrezza Belluomini Castro, Rafaela Aparecida Prata, Marla Andréia Garcia de Avila, Milena Temer Jamas

**Affiliations:** 1 Universidade Estadual Paulista, Departamento de Enfermagem, Botucatu, SP, Brasil.

**Keywords:** Enfermería Obstétrica, Miedo, Parto, Internet, Educación para la Salud, Persona Embarazada

## Abstract

**Objetivo:**

identificar la prevalencia y los factores asociados con el miedo al parto en adultos brasileños.

**Método:**

estudio transversal con 528 participantes de entre 18 y 58 años. Los datos se recopilaron mediante una encuesta en línea, que abordó características sociodemográficas y conductuales, así como información relacionada con la salud, la historia obstétrica y el miedo al parto, según la versión brasileña del instrumento *Childbirth Fear Before to Pregnancy*. Las variables fueron analizadas y ajustadas por múltiples modelos de regresión lineal, asumiendo que los residuos siguen una distribución normal.

**Resultados:**

la prevalencia del miedo al parto fue del 28,4% (n=133) para el bajo miedo, del 19,5% (n=103) para el moderado y del 26,9% (n=142) para el alto miedo al parto. Los análisis multivariantes mostraron que puntuaciones más bajas de miedo al parto se asociaban con una edad más joven (p=0,002) y el sexo femenino (p<0,000). Por otro lado, las personas con embarazos previos obtuvieron puntuaciones CPFF más altas (p=0,005).

**Conclusión:**

la prevalencia del miedo al parto fue del 71,6% (n=378), distribuida entre un miedo bajo (25,2%), moderado (19,5%) y alto (26,9%). Factores como la edad avanzada y el género femenino se asociaron con puntuaciones de miedo más bajas, mientras que el embarazo previo se asoció con un nivel más alto de miedo. Estos resultados sugieren que la experiencia de la madre puede influir en decisiones futuras sobre la elección de su propio nacimiento o influir en la percepción que otras personas tienen de su vida.

## Destacados

**(1)** Las puntuaciones más altas por miedo al parto se asociaron con el género femenino. **(2)** Prevalencia del miedo al parto en la población que quiere tener hijos. **(3)** La población más joven tiene más miedo al parto. **(4)** Mayor asociación con el miedo al parto en mujeres con experiencia previa.

## Introducción

El miedo al parto se caracteriza por una sensación de inseguridad y ansiedad antes, durante o después del parto, en relación con el proceso de parto y el momento del parto, con variaciones de niveles más leves o más graves^1^ ^-^ ^2^. Este sentimiento puede deberse a informes o malas experiencias en partos anteriores generadas por el trato hostil por parte del equipo, a una sensación de abandono o no participación en las decisiones sobre el cuidado^3^ ^-^ ^4^.

El miedo intenso al parto es un problema multifacético que va mucho más allá del propio evento obstétrico, afectando significativamente la salud mental y el bienestar emocional de las mujeres a largo plazo. Como demuestra la literatura, la complejidad del parto, que moviliza una serie de sentimientos contradictorios como la ansiedad, la inseguridad y el estrés, ya apunta a la relevancia de los aspectos psicológicos implicados^5^.

Las evidencias científicas indican que los factores socioculturales juegan un papel importante en desencadenar el miedo al parto. Las narrativas culturales, transmitidas por familiares y amigos, que asocian el parto vaginal con dolor y sufrimiento, contribuyen a que las mujeres desarrollen ansiedad, inseguridad y miedos aumentados. Esto resulta en la subestimación de la propia capacidad fisiológica para dar a luz, a pesar de la historia milenaria de partos naturales sin medicalización. El gran desafío en la atención obstétrica es, por tanto, devolver el control sobre su cuerpo y pensamientos a la mujer embarazada, promoviendo esta confianza durante todo el periodo prenatal, y no solo en las horas previas al parto^6^.

Esta profunda aprensión puede generar una serie de consecuencias que se manifiestan de diferentes maneras en la vida de las mujeres. Además de los efectos fisiológicos ya conocidos - como dilatación prolongada, mayor riesgo de preeclampsia, parto prematuro y cesárea de urgencia - el miedo al parto está intrínsecamente ligado a resultados negativos en la salud mental y en la calidad de vida. Puede provocar el desarrollo de depresión posparto, afectar las tasas de lactancia materna e incluso resultar en una mayor necesidad de ingreso del recién nacido en unidades de cuidados intensivos^7^ ^-^ ^8^.

Este estudio identificó que el primer embarazo, el nivel educativo más bajo, la concepción natural y la preferencia por la cesárea están asociados a un mayor nivel de miedo. Además, se identificaron comorbilidades como la miopía alta y las enfermedades del tracto urinario como predictores independientes del miedo severo. Reconocer la complejidad del miedo al parto es fundamental para los profesionales sanitarios que integran el cribado obstétrico en la atención prenatal, especialmente en grupos de riesgo. Esto permite asesoramiento y apoyo personalizados, promoviendo decisiones informadas en el parto y contribuyendo al bienestar psicológico materno y a mejores resultados obstétricos^9^.

Suecia es uno de los pocos países donde el miedo al parto se reconoce como un problema importante y donde la mayoría de las mujeres embarazadas ofrecen servicios de asesoramiento. Una encuesta realizada en 2006 con 2.262 mujeres suecas indicó que la prevalencia del miedo al parto rondaba el 10%. Las mujeres fueron reclutadas en su primera visita a una clínica prenatal durante tres semanas predeterminadas repartidas a lo largo de un año. El estudio reveló que el miedo al parto estaba asociado con un aumento en la tasa de cesáreas electivas y con una experiencia de parto “aceptable” en mujeres que recibieron asesoramiento^4^.

En un estudio realizado en Croacia, con estudiantes universitarios jóvenes y no embarazados de las carreras de ciencias de la salud, ciencias sociales y humanidades, se demostró que el 25,9% de los estudiantes reportaba un miedo significativo al parto, y que el de los estudiantes de ciencias de la salud estaba en un nivel inferior en comparación con los otros estudiantes de los otros programas, además de haber recibido más información sobre el parto en el contexto familiar en comparación con el proceso de aprendizaje. Se cree que el nivel más alto de miedo al parto podría predecirse en la identificación de factores como la sensibilidad a la ansiedad, el rasgo de ansiedad, la expectativa de dolor de parto y fuentes de conocimiento sobre el parto^10^.

El miedo al parto afecta negativamente a las mujeres de varias maneras, lo que puede desencadenar un aumento en el uso de analgesia de parto, desequilibrio emocional, prolongación del parto, la elección de cesárea electiva, dificultad para vincularse con el bebé y depresión posparto^11^ ^-^ ^14^. Además, esto puede afectar la armonía en las relaciones familiares y de pareja. Existen pruebas sustanciales que indican que altos niveles de miedo al parto conducen a trastorno de estrés postraumático, ansiedad posparto, depresión y psicosis, e incluso aumentan la necesidad de cuidados psiquiátricos a largo plazo^15^ ^-^ ^16^.

Cada vez hay más evidencias que sugieren que el miedo al parto no es una prerrogativa exclusiva de las mujeres; los futuros padres también pueden experimentar ansiedades significativas relacionadas con el parto, aunque sus preocupaciones y manifestaciones pueden variar. La inclusión de la perspectiva masculina en la investigación del miedo al parto es fundamental, no solo para comprender la experiencia paterna en sí, sino también por el impacto que el bienestar del padre puede tener en la pareja, en la dinámica familiar y en el proceso de transición hacia la parentalidad^17^. Ignorar el miedo al parto en los hombres significa dejar a una parte de la población sin el apoyo y la comprensión necesarios durante un periodo de vulnerabilidad.

La adopción de medidas de evaluación e intervención en el parto contribuye a desmitificar los miedos y creencias erróneas, mejorando el bienestar y la calidad de vida de los jóvenes, además de reforzar los esfuerzos internacionales para reducir las tasas de cesáreas electivas y la medicalización. Es importante evaluar e intervenir previamente en poblaciones no embarazadas, ya que es durante el periodo gestacional cuando los miedos se intensifican^18^.

En este escenario, la enfermería asume un papel central e insustituible. Las enfermeras especialistas, con su proximidad y enfoque holístico de la atención perinatal, se encuentran en una posición privilegiada para actuar como facilitadoras en el proceso de desmitificar el parto y reducir el miedo asociado. A través de la escucha activa, la educación perinatal integral y la implementación de prácticas de atención personalizadas y basadas en la evidencia, la enfermería puede empoderar tanto a mujeres como a hombres. Esto implica desmontar mitos, proporcionar información clara y realista sobre el proceso de parto, debatir opciones para el manejo del dolor, promover el protagonismo de la pareja y crear un ambiente de confianza y seguridad^19^ ^-^ ^21^.

Un estudio cualitativo^22^ investigó la percepción de las mujeres sobre la terapia de comadronas en relación con el miedo al parto. Los resultados revelaron una mejora en la confianza de las mujeres en el parto tras adquirir información y conocimientos. Las participantes informaron de una mayor calma y preparación, lo que fortaleció su capacidad para afrontar la incertidumbre del proceso de parto, impactando positivamente en la experiencia del parto. Además, la sensación de seguridad que proporciona el apoyo profesional durante el parto contribuyó a que las mujeres se sintieran empoderadas. Esta experiencia positiva, a su vez, reforzó la autoconfianza para futuros partos, y el miedo al parto se percibió como reducido o controlable.

A pesar de su eficacia probada para reducir el dolor, disminuir el tiempo de parto y el parto en sí, y debido a que son de bajo coste y fáciles de aplicar, métodos no farmacológicos como la terapia de calor, masajes, aromaterapia, acupresión, danza y ejercicios de pelota suiza siguen siendo poco publicitados. Estos enfoques también se asocian con un menor uso de intervenciones farmacológicas para la analgesia. Sin embargo, es en el escenario del parto, con la presencia y el rendimiento de la enfermería obstétrica, donde estos métodos ganan mayor visibilidad y se utilizan más ampliamente^23^ ^-^ ^24^. La enfermería desempeña un papel crucial como facilitadora en la desmitificación del parto y la reducción del miedo al promover activamente estas prácticas de atención personalizadas y basadas en la evidencia que empoderan a las mujeres y contribuyen a una experiencia del parto más positiva y menos medicalizada.

Es imprescindible investigar los factores asociados con el miedo al parto, ampliando el ámbito tradicional para incluir a la población masculina y considerando la evaluación en fases previas al embarazo, especialmente en el contexto brasileño. Un conocimiento profundo de estos factores es esencial para apoyar el desarrollo de estrategias de intervención más eficaces, inclusivas y culturalmente sensibles, especialmente promovidas por la enfermería, con el objetivo de mejorar la salud perinatal y la experiencia de parto para todos los implicados. Por ello, este estudio buscó identificar la prevalencia y los factores asociados con el miedo al parto en adultos brasileños.

## Método

### Diseño del estudio

Se trata de un estudio transversal y analítico, realizado mediante una encuesta en línea a adultos mayores de 18 años, brasileños de las cinco macroregiones del país, guiado por la directriz *Strengthening the Reporting of Observational Studies in Epidemiology* (STROBE)^25^, realizada entre enero y abril de 2022 a través del formulario digital disponible por la herramienta Google *Forms*.

### Definición de la muestra

El estudio incluyó a hombres y mujeres mayores de 18 años, independientemente de su estado civil, con la intención de tener hijos en el futuro. Se excluyeron los participantes que no respondieron al instrumento durante el periodo de recogida de datos y aquellos cuyos formularios no tenían todas las preguntas respondidas.

Teniendo en cuenta la población objetivo, formada por adultos mayores de 18 años que pretendían tener hijos en el futuro, residentes en cualquier región del país, la muestra se clasifica como no probabilística del tipo intencional. Es prudente considerar que el plan de muestreo adoptado, de muestreo intencionado no probabilístico de 528 participantes, puede generar errores sistemáticos de selección que influyen en las estimaciones de las asociaciones entre los factores investigados y el resultado de la puntuación por miedo al parto, especialmente si consideramos que la intención es inferir la población brasileña.

Sin embargo, para estimar su tamaño, se consideró que la intención era conocer un proceso y no inferirlo para una población específicamente delimitada en espacio-tiempo. Así, para el cálculo del número mínimo de participantes (y no para el tamaño mínimo de una muestra representativa de una población objetivo), se asumió una prevalencia de 0,50, un nivel de significancia del 5% y una precisión del 3%.

### Variables del estudio

Las variables independientes eran: datos sociodemográficos como la edad (en años); género (masculino; femenino; prefiero no identificarme); color de piel autodeclarado (blanco; negro; marrón; autóctono; amarillo); educación (sin escolarización; escuela primaria incompleta; escuela primaria completa; secundaria incompleta; educación secundaria completa; educación superior; posgrado); origen (norte; noreste; sur; sureste; centro-oeste); profesional de la salud (sí o no); estado civil (con o sin pareja). Variables obstétricas: tiene hijos (sí o no); tipo de parto del último embarazo (normal; cesárea); tuvo una experiencia negativa en el embarazo anterior (sí; no); variables conductuales: las experiencias de amigos o familiares influyeron en el tipo de parto que eligieron (sí; no); fuente de información de la población estudiada: dónde buscan información sobre el parto (vídeos/internet; material impreso; familiares y amigos; profesionales de la salud; artículos científicos; no busco información sobre el parto). En este estudio, se consideró la escala *Childbirth Fear Prior to Pregnancy* (CFPP) como una variable dependiente, como instrumento para evaluar el miedo al parto, ya que permite que la evaluación del miedo antes de la concepción, tanto en hombres como en mujeres, sea reconocida por su lenguaje accesible, fácil de aplicar y que ayuda al desarrollo de intervenciones específicas. Sus dimensiones abordan el miedo al dolor de parto, el miedo a complicaciones relacionadas con el bebé y el miedo a lesiones corporales^18^.

### Recogida de datos

Para este estudio, los investigadores desarrollaron un instrumento virtual de recogida de datos utilizando la plataforma Google *Forms*. Este cuestionario constaba de 26 preguntas cerradas para recopilar información sobre las características sociodemográficas, obstétricas y conductuales de los participantes (variables independientes).

La versión brasileña en línea de la CFPP fue adoptada como instrumento para medir la variable dependiente, ya que es el único instrumento validado para la población brasileña en la evaluación del miedo al parto en hombres y mujeres^26^. Otro punto relevante son sus características psicométricas adecuadas, incluyendo un lenguaje claro y fácil de entender, lo que facilita su aplicación. Estas cualidades convierten al CFPP en una herramienta valiosa no solo para la investigación, sino también para el desarrollo de intervenciones efectivas destinadas a reducir el miedo al parto^18^.

La versión brasileña en línea del CFPP es un instrumento corto y sencillo, unidimensional, con 10 ítems, con 5 opciones de respuesta que van desde (1) estar en fuerte desacuerdo, (2) no estar de acuerdo, (3) estar parcialmente en desacuerdo, (4) estar de acuerdo y (5) totalmente de acuerdo, dispuestos verticalmente. La puntuación final se obtiene sumando las puntuaciones de los 10 ítems, con una puntuación mínima de 10 y un máximo de 50^26^.

La recopilación de datos se realizó mediante una encuesta en línea, siguiendo las recomendaciones de la lista CHERRIES (*Checklist for Reporting Results of Internet E-Surveys*)^27^, lo que permitió, en un momento en que el distanciamiento social requería la necesidad de mantener la distancia, llegar a una muestra diversificada a través de internet, cubriendo a individuos en diferentes regiones geográficas de Brasil y de forma inmediata^28^.

La recopilación de datos fue realizada por los investigadores y tres estudiantes de grado, colaboradores de la investigación. Los participantes fueron reclutados mediante la difusión del formulario digital, disponible a través de Google *Forms*, en varios canales online, incluyendo correos electrónicos, redes sociales (Facebook e Instagram) y contactos personales a través de WhatsApp. Este proceso se expandió mediante un efecto multiplicador, llamado *bola de neve* (“*snowball*” - bola de nieve) o muestreo de seguimiento de enlaces. El muestreo en bola de nieve es una técnica no probabilística que genera un efecto multiplicador en la recogida de datos, similar al crecimiento de una bola de nieve. El proceso comienza con la identificación de uno o más “informantes clave”. Estos, a su vez, indican nuevos participantes en sus redes de contactos que cumplen con los criterios de investigación, creando una cadena de referencias que se expande exponencialmente. Este enfoque es especialmente eficaz para acceder a grupos difíciles de alcanzar o que abordan temas sensibles, ya que aprovecha las conexiones existentes entre individuos^29^.

### Análisis de datos

Los datos recopilados se transportaron y organizaron en el programa Microsoft Office Excel. Los análisis estadísticos se realizaron utilizando el *software* IBM - *Statistical Package for the Social Sciences* (SPSS versión 21).

Primeramente, se realizó un análisis exploratorio de los datos para identificar y tratar valores atípicos y identificar el mecanismo de generación de ausencias. Tras la evaluación inicial, se realizó un análisis bivariado para investigar la asociación aislada de cada variable independiente con la puntuación CFPP mediante regresiones lineales simples con una respuesta con una distribución probabilística normal (que acomoda resultados numéricos con al menos un grado razonable de simetría).

El corte p<0,20 se utiliza para evitar omitir una variable que tenga un papel importante y consolidado dentro de un modelo de determinación social o dentro de la patogénesis del resultado solo por una asociación más débil. En otras palabras, prioriza los paradigmas de salud en lugar de un criterio estadístico. En el modelo de regresión múltiple, las asociaciones se consideraron estadísticamente significativas si p < 0,05.

### Aspectos éticos

Todas las actividades se desarrollaron tras la aprobación del Comité de Ética en la Investigación de la Facultad de Medicina de Botucatu, bajo el dictamen n.º 4.836.471, de acuerdo con la Resolución del Consejo Nacional de Salud n.º 510/2016. La aceptación para participar en la investigación se registró a través del Formulario de Consentimiento Informado (FCI) en formato digital^30^.

## Resultados

Un total de 671 personas aceptaron participar en el estudio. Sin embargo, 143 participantes fueron excluidos porque no cumplían los criterios de inclusión, siendo el estudio la muestra final de 528 participantes de las cinco macroregiones de Brasil.

De los participantes, el 81,6% (n=431) eran mujeres, con una mediana de 24 años (mínimo de 18 y máximo de 58 años), el 69,3% (n=366) se declaró blanco o amarillo, el 75,8% (n=400) con al menos el nivel de secundaria, el 50,9% (n=269) eran profesionales de la salud, y el 61% (n=322) reportó tener pareja. La región del Sureste fue la más representada, con un 62,1% (n=328) de los participantes, como se muestra en la Tabla 1.

**Tabla 1:** Caracterización sociodemográfica de los participantes (n = 528). Brasil, 2024

Variables	Total	
	n*	%^†^
**Género**		
Femenino	431	81,6
Masculino	96	18,2
No identificó	1	0,2
**Raza/Color**		
Blanco/Amarillo	366	69,3
Negro/Marrón/Indígena	162	30,7
**Educación**		
Educación primaria y secundaria incompletas	128	24,2
Educación secundaria, grado y posgrado	400	75,8
**Estado civil**		
Con la pareja	322	61,0
Sin compañero	206	39,0
**Región**		
Sureste	328	62,1
Medio Oeste	83	15,7
Noreste	57	10,8
Sur	35	6,6
Norte	25	4,7
**Profesional de la salud**		
Sí	269	50,9
No	259	49,1

*n = Número de participantes; ^†^% = Porcentaje

De las 95 participantes que informaron tener hijos, el 29,4% (n=28) ya había tenido experiencia con parto vaginal o lo había experimentado con sus parejas. Además, el 33 (6,3%) de las participantes reportaron experiencias negativas en el último embarazo y el 71,6% (n=378) reportó miedo al parto. La búsqueda de información sobre el parto fue reportada por el 74,6% (n=394), con un 26,3% (n=139) que buscó esta información principalmente de profesionales sanitarios y un 22,2% (n=117) en internet y vídeos, como se muestra en la Tabla 22.

**Tabla 2:** Características obstétricas y percepciones del parto y fuente de información entre adultos. Brasil, 2024

Variables	n*	%^†^
Tiene hijos (n=528)	95	18,0
Parto vaginal en el último embarazo (Sí) (n=95)	28	29,5
Experiencia negativa en el último embarazo (Sí) (n=95)	33	34,7
Experiencias de amigos y familiares influyen en la elección del tipo de parto (Sí) (n=528)	123	23,3
Miedo al parto (Sí) (n=528)	378	71,6
Busca informaciones sobre el parto (Sí) (n=528)	394	74,6
**Lugares donde buscan las informaciones**		
Profesional de la salud	139	26,3
Vídeos/internet	117	22,2
Artículos científicos	79	15,0
Familiares y amigos	54	10,2
Material impreso	05	0,9

Variables	n*	%^†^
Tiene hijos (n=528)	95	18,0
Parto vaginal en el último embarazo (Sí) (n=95)	28	29,5
Experiencia negativa en el último embarazo (Sí) (n=95)	33	34,7
Experiencias de amigos y familiares influyen en la elección del tipo de parto (Sí) (n=528)	123	23,3
Miedo al parto (Sí) (n=528)	378	71,6
Busca informaciones sobre el parto (Sí) (n=528)	394	74,6
**Lugares donde buscan las informaciones**		
Profesional de la salud	139	26,3
Vídeos/internet	117	22,2
Artículos científicos	79	15,0
Familiares y amigos	54	10,2
Material impreso	05	0,9

*n = Número de participantes; ^†^% = Porcentaje

La Tabla 3 muestra las puntuaciones de las participantes por miedo al parto, siguiendo la clasificación CFPP. La prevalencia del miedo al parto fue del 71,6% (n=378), con un 25,2% (n=133) bajo, un 19,5% (n=103) moderado y un 26,9% (n=142) alto miedo al parto.

**Tabla 3:** Clasificación del miedo al parto en adultos según CFPP* (n = 528). Brasil, 2024

Clasificación	Puntuación CFPP*	n^†^	%^‡^
Ausencia de miedo	10 ≤ x ≤ 28	150	28,4
Bajo miedo	29 ≤ x ≤ 33	133	25,2
Miedo moderado	34 ≤ x ≤ 37	103	19,5
Alto miedo	38 ≤ x ≤ 50	142	26,9

*Versión de la escala *Childbirth Fear Prior to Pregnancy* con una puntuación de 10 a 50; ^†^n = Número de participantes; ^‡^% = Porcentaje

Según los resultados presentados en la Tabla 4, el análisis bivariado investigó la asociación entre variables independientes y la puntuación CFPP. Las asociaciones estadísticamente significativas (p < 0,20) fueron las siguientes variables: edad (p < 0,001), género femenino (p < 0,001), no ser profesional sanitario (p = 0,027), vivir en el Sureste (p=0,130) y el Noreste (p=0,150), estado civil con pareja (p=0,137), no tener hijos (0,051), tener más de un embarazo (p=0,001), experiencia con amigos/familiares, elección del tipo de parto (p=0,017), Búsqueda de información en artículos científicos (p= 0,035), familiares y amigos (p=0,027), profesionales de la salud (p=0,007) y vídeos/internet (p=0,007).

**Tabla 4:** Asociación bivariante entre variables sociodemográficas, obstétricas y conductuales y la puntuación CFPP* (n = 528). Brasil, 2024

Variables	b^†^	IC^‡^95%		*p* ^*§*^
		Límite Inferior	Límite Superior	
Edad	-0,17	-,25	-,08	**0,000**
Género				
Masculino	3,80	-10,95	18,56	0,614
Femenino	-3,48	-5,14	-1,81	**0,000**
Otra clasificación^||^				
Raza o Color				
Blanco/Amarillo	0,32	-1,09	1,74	0,654
Negro/Marrón/Indígena^¶^				
Región
Medio Oeste	-2,35	-6,23	1,54	0,236
Sureste	2,46	-,72	5,65	**0,130**
Sur	1,18	-1,81	4,17	0,441
Noreste	1,94	-,70	4,58	**0,150**
Norte**				
No ser profesional de la salud	-1,47	-2,77	-0,17	**0,027**
Trabajo de parto	-1,89	-4,85	1,06	0,210
Educación mínima educación secundaria	0,23	-1,29	1,75	0,768
Estado civil con la pareja	-1,01	-2,35	0,32	**0,137**
No tener hijos	-1,69	-3,38	0,00	**0,051**
Tener más de un embarazo	4,94	2,06	7,82	**0,001**
Experiencia negativa en el último embarazo	-0,22	-2,91	2,47	0,871
Experiencia de amigos/familiares elección del tipo de parto	1,08	-,46	2,62	**0,170**
Buscar informaciones sobre el parto	-0,30	-1,80	1,19	0,691
Lugares que buscan informaciones				
Artículos científicos	-1,95	-3,77	-0,13	**0,035**
Materiales impresos	-1,39	-8,11	5,34	0,687
Familiares, amigos	2,42	,27	4,56	**0,027**
Profesionales de la Salud	-2,01	-3,48	-,54	**0,007**
Vídeo/Internet	2,15	,59	3,71	**0,007**

*Versión de la escala *Childbirth Fear Prior to Pregnancy;* ^*†*^ Intercepto; ^‡^Intervalo de confianza; ^§^Análisis bivariado mediante regresión lineal simple; ^||^Referencia para género; ^¶^Referencia para color/raza; **Referencia para regiones en Brasil

Se realizó un análisis multivariante mediante regresión lineal múltiple, con la puntuación total de la escala CFPP como variable dependiente (puntuación que va de 10 a 50). Los resultados mostraron que cuanto mayor es la edad, menor es la puntuación obtenida en la escala, es decir, menor es el miedo al parto (b = -0,17; IC 95% -0,25 a -0,08; p < 0,001). Además, los hombres obtuvieron una puntuación media 3,6 puntos más baja que las mujeres, mostrando menos miedo al parto (b = -3,48; IC 95% de -5,14 a -1,81; p < 0,001). Por otro lado, los participantes con experiencia gestacional previa con puntuaciones más altas se asociaron con los que reportaron experiencia en parto; presentó una media de 4,5 puntos más, mostrando un mayor miedo al parto (b = 4,52; IC 95%: 1,35 a 7,68; p = 0,005) (Tabla 5).

**Tabla 5:** Regresión lineal múltiple entre variables sociodemográficas, obstétricas y conductuales, y la puntuación total de la escala CFPP* (n = 528). Brasil, 2024

Variables	b^†^	IC^‡^95%		*p* ^*§*^
		Límite Inferior	Límite Superior	
Edad	-0,16	-,26	-,06	**0,002**
Género				
Masculino	1,87	-12,22	15,96	0,795
Femenino	-3,68	-5,32	-2,04	**0,000**
Otra clasificación^||^				
Región
Medio Oeste	-1,89	-5,65	1,87	0,324
Sureste	0,97	-2,09	4,03	0,534
Sur	0,77	-2,09	3,64	0,597
Noreste	1,17	-1,37	3,71	0,365
Norte^¶^				
No ser profesional de la salud	-1,22	-2,54	0,11	0,072
Estado civil con la pareja	0,13	-1,25	1,50	0,855
No tener hijos	1,26	-,98	3,50	0,271
Tener más de un embarazo	4,52	1,35	7,68	**0,005**
Experiencia de amigos/familiares elección del tipo de parto	1,45	-,07	2,97	0,062
Buscan informaciones con familiares, amigos	1,58	-0,76	3,93	0,186
Buscan informaciones en artículos científicos	1,60	-3,70	,50	0,134
Buscan informaciones con profesionales de la salud	-1,43	-3,18	,33	0,110
Buscan informaciones con vídeo/internet	0,82	-,99	2,62	0,376

*Versión de la escala *Childbirth Fear Prior to Pregnancy;* ^*†*^ Intercepto; ^‡^Intervalo de confianza; ^§^Análisis bivariado mediante regresión lineal múltiple; ^||^Referencia para género; ^¶^Referencia para regiones en Brasil

## Discusión

El destaque del presente estudio fue buscar evidencias, a través de un instrumento apropiado al contexto brasileño, que pudieran contribuir a la identificación de factores asociados con el temor al parto normal en la población brasileña, incluidos hombres y mujeres, proporcionando subsidios para la realización de materiales educativos y acciones políticas sobre el tema.

La tocofobia, o miedo intenso al parto, afecta aproximadamente al 14% de las mujeres a nivel global^11^. Al analizar la prevalencia en diferentes países, se observa una variación significativa. En Estados Unidos, por ejemplo, la condición es más frecuente, afectando al 26,9% de las mujeres embarazadas^31^. En países como Australia y Finlandia, la tasa de miedo al parto está entre el 20% y el 25%^32^ ^-^ ^33^. Estos datos destacan la importancia de comprender y abordar la tocofobia, teniendo en cuenta sus particularidades culturales y geográficas.

Un estudio cualitativo realizado en el Reino Unido, que incluyó a 10 mujeres embarazadas y 13 parteras, identificó elementos importantes que influyen en el miedo al parto, como el miedo a no poder afrontar lo impredecible; temor a que el bebé o la propia mujer embarazada resulten heridos durante el parto; miedo a no poder dar a luz; miedo a no saber cómo manejar el dolor; miedo a no tener autonomía para tomar decisiones; miedo a intervenciones; miedo al abandono y miedo a perder el control. En este estudio, cuando se les preguntó cuándo deberían hacerse preguntas sobre el miedo al parto, la mayoría respondió lo antes posible. Destacó que es esencial evaluar el miedo al parto en las mujeres embarazadas lo antes posible, indicando hasta la semana 20 de gestación. La evaluación temprana permite identificar e intervenir de manera eficaz, reduciendo la angustia de las mujeres y minimizando el miedo al parto antes del parto^34^.

En el presente estudio, las experiencias previas de embarazo mostraron una asociación con el miedo al parto, en línea con los resultados de la metasíntesis, que incluyó 14 estudios cualitativos y buscó comprender las experiencias de mujeres con miedo al parto^28^. El estudio muestra que las mujeres sufrieron consecuencias por experiencias traumáticas de parto, pensamientos sobre partos anteriores o al escuchar a otras mujeres contar su terrible experiencia de parto. Los autores señalan que la mayoría de las mujeres multiparas no sentían miedo durante su primer embarazo, pero tras haber experimentado un parto traumático, estaban aterrorizadas por la experiencia de otro parto. Recomiendan que las mujeres que tienen miedo tras una experiencia negativa previa necesiten apoyo para poder recuperar la confianza en los profesionales de la maternidad. Las primigestas requieren un apoyo similar para asegurar que las experiencias de otras mujeres no se repitan con ellas^35^. Se reconoce que los profesionales de la salud son mensajeros clave que pueden mejorar o empeorar el miedo al parto en las mujeres, dependiendo del modo de atención proporcionado^34^.

Un estudio cualitativo realizado en Brasil señala los mitos y miedos que impregnan el parto y destaca que tanto las mujeres embarazadas como las puérperas verbalizaban el dolor como un factor que podría impedir la preferencia por un parto normal. Las autoras consideran que el alto grado atribuido a la experiencia del parto en la vida de las mujeres, que deja huellas en sus espaldas, justifica la urgente necesidad de revisar las prácticas e interacciones inhumanas que aún están presentes en la postura de tantos profesionales, sustituyéndolas por interacciones armoniosas^36^.

Una revisión sistemática que incluyó 21 estudios tuvo como objetivo identificar posibles causas y factores predisponentes y resultados del miedo al parto en mujeres en edad fértil. En el estudio, el factor predictivo más fuerte para el miedo al parto en mujeres multíparas fue la experiencia negativa del parto vaginal o cesárea. Factores como el parto prolongado, el uso de epidurales, complicaciones obstétricas, la presencia de síntomas de estrés traumático y la necesidad de cuidados psiquiátricos son resultados reportados en mujeres con alto miedo al parto^16^.

En una revisión bibliográfica realizada por investigadores brasileños^37^, con el objetivo de revisar conceptos y definiciones sobre el miedo al parto, los hallazgos fueron diferentes de los encontrados en nuestro estudio. La revisión señala que las mujeres nulíparas tienen más miedo al parto que las multíparas, tanto al principio como al final del embarazo, y que la edad gestacional avanzada se asocia con un mayor nivel de miedo al parto. Los resultados demostraron que el miedo al parto está relacionado con un mayor riesgo de eventos obstétricos adversos, como el aumento de cesáreas, parto prematuro, parto prolongado, depresión posparto y estrés postraumático. Esta evidencia pone de relieve la importancia de hablar sobre el miedo al parto prenatal y alerta sobre la necesidad de futuras estrategias para la evaluación y tratamiento de este miedo^37^.

En nuestro estudio, ser más joven, especialmente menor de 25,5 años, fue un factor que influyó en el miedo al parto. Nuestros hallazgos corroboran diferentes estudios. Investigaciones realizadas en Estados Unidos, que incluyen a 758 jóvenes y estudiantes, sugieren que las mujeres tienen más déficits de conocimiento y quieren aprender más sobre el parto. El estudio también identificó que los canales informales, como los amigos y la familia, ejercen una gran influencia en las decisiones de las jóvenes en relación con el parto^14^.

Los factores sociodemográficos pueden tener poca influencia en las intervenciones sanitarias, pero es esencial considerar su influencia en las condiciones de las personas al planificar la atención al parto. Contrariamente a nuestros hallazgos, una revisión sistemática no identificó una asociación entre el miedo al parto y la edad. El impacto de la edad en el miedo al parto varió entre estudios: algunos no encontraron asociación, mientras que otros identificaron un mayor riesgo en mujeres nulíparas < 32 años y en mujeres nulíparas y multiparas ≥ 40 años. Además, la variación en el tamaño de las poblaciones estudiadas puede tener implicaciones para los resultados^16^.

Por esta razón, se recomienda que la educación sobre el parto se realice mucho antes del primer parto de una joven, cuando se están estableciendo opiniones sobre la atención materna y existe el potencial de reforzar la idea de que un parto de baja intervención, seguido de un parto vaginal, es la opción más saludable y mejor para la mayoría de las mujeres en edad fértil^37^.

Un estudio transversal, que pretendió rastrear el perfil epidemiológico del miedo al parto en mujeres embarazadas brasileñas, utilizó la versión portuguesa del *Wijma Delivery Expectancy/Experience Questionnaire* (W-DEQ) y demostró una correlación positiva débil entre la edad de la mujer embarazada y la puntuación (W-DEQ), sugiriendo que cuanto mayor es la mujer, mayores son sus niveles de miedo al parto^13^.

Nuestro estudio evaluó el miedo a hombres y mujeres, junto con el miedo asociado al género femenino. Aunque el miedo al parto suele ser más debatido entre las mujeres, es fundamental reconocer que la figura paterna puede ser un apoyo fundamental para la mujer embarazada. Muchos padres se convierten en el principal apoyo emocional y práctico durante el embarazo y el parto.

Una revisión integradora examinó y sintetizó los resultados de 17 encuestas relacionadas con el miedo al parto paterno e identificó que los futuros padres experimentan un miedo patológico al parto, relacionado con la salud y vida del bebé, la salud y la vida de la pareja, así como sus propias reacciones y comportamientos durante el proceso. Esta investigación reveló que el miedo al parto influye negativamente en la vida de los hombres y, en consecuencia, en la de sus familias. El estudio destaca la necesidad de investigar más a fondo métodos y modelos para identificar el miedo paterno, utilizando instrumentos fiables validados para la cultura local, dirigidos a hombres en riesgo o con miedo al parto. Además, es esencial desarrollar nuevas estrategias educativas sobre el parto, proporcionando información adecuada para esta población, ya que el miedo paterno al parto puede influir en la elección de las mujeres por la cesárea. Una intervención que incluya a la pareja puede ser apropiada en algunos casos^17^.

Un estudio realizado en el Reino Unido con estudiantes sin hijos para evaluar la influencia de las representaciones socioculturales y las percepciones sobre el parto reveló que las estudiantes con impresiones negativas sobre el parto tenían una probabilidad significativamente mayor de tener puntuaciones más altas de miedo al parto. Las impresiones negativas del embarazo percibidas en amigos o familiares y las percepciones del nacimiento representadas en medios visuales se asociaron con mayores puntuaciones de miedo al parto. Las estudiantes que tenían experiencia asistiendo a un parto describieron la experiencia como increíble, y sus puntuaciones fueron más bajas por miedo al parto. Estos hallazgos ponen de manifiesto la posible influencia negativa de las representaciones socioculturales. También indican la necesidad de estudios de métodos mixtos para determinar los efectos positivos de promover imágenes y mensajes positivos sobre el parto y el parto dentro de las iniciativas de salud pública^38^.

Incluso con los hallazgos de este estudio que apuntan al miedo al parto predominantemente en mujeres, se entiende que las intervenciones para el cuidado del parto también deben dirigirse a los hombres, ya que también están afectados por los componentes del embarazo. Un estudio realizado con una muestra de hombres australianos para identificar los factores de riesgo asociados al malestar mental perinatal paterno encontró que los trastornos del sueño, el malestar mental debido a embarazos no planeados, el conflicto trabajo-familia, el estrés relacionado con el papel de proveedor, el malestar matrimonial y la depresión materna son factores de riesgo significativos que afectan a los hombres y aumentan el malestar mental paterno durante el embarazo. Estos hallazgos son de gran importancia para el desarrollo de intervenciones tempranas específicas para la atención perinatal paterna^39^.

En este estudio, el 46,5% de los participantes eran profesionales sanitarios que mostraron miedo respecto al parto. Es importante destacar que la presencia del miedo puede crear obstáculos para estos profesionales a la hora de desmitificar los sentimientos negativos que rodean el proceso de parto. Es fundamental que estos profesionales reconozcan y aborden sus propias preocupaciones sobre el parto para garantizar una atención adecuada y acogedora a las mujeres embarazadas.

Como limitaciones de esta investigación, se destaca la menor participación de individuos masculinos, lo que pudo haber influido en el análisis comparativo entre géneros. Además, la selección se llevó a cabo en un entorno virtual, lo que pudo haber excluido a personas en situaciones de vulnerabilidad social, con acceso limitado a internet, equipos electrónicos o con baja competencia digital. También cabe destacar que la muestra se obtuvo mediante un método no probabilístico del tipo intencional, lo que representa una limitación metodológica, ya que restringe la posibilidad de generalizar los hallazgos a la población brasileña en su conjunto. Aunque el objetivo del estudio no era inferencial, esta característica debe tenerse en cuenta en la interpretación de los resultados.

En resumen, este estudio revela que el miedo al parto es una realidad expresiva entre los adultos brasileños, alcanzando una prevalencia considerable. Se pone de manifiesto la complejidad de este fenómeno, con la influencia de factores como la edad y la experiencia gestacional previa en la intensidad del miedo. La identificación de estos predictores es crucial, ya que ofrece valiosos subsidios para el desarrollo de estrategias de salud pública más efectivas. Comprender quiénes son los grupos más vulnerables al miedo al parto permite crear intervenciones específicas, con el objetivo de ofrecer apoyo psicológico y educativo, que no solo puede mejorar la experiencia del parto, sino que también tiene un impacto positivo en las decisiones reproductivas y en la salud mental de la población general.

## Conclusión

Observou-se que o medo do parto é frequente entre adultos brasileiros. Os escores mais altos do medo do parto mostraram-se associados a adultos, ao sexo feminino e a mulheres que já haviam tido uma gestação. As variáveis que exercem influência no medo do parto devem ser valorizadas nas intervenções de saúde, antes do início da gestação e durante o pré-natal. Se observó que el miedo al parto es frecuente entre los adultos brasileños. Las puntuaciones más altas por miedo al parto se encontraron asociadas a adultos, mujeres y mujeres que ya habían tenido un embarazo. Las variables que influyen en el miedo al parto deben valorarse en las intervenciones sanitarias, antes del inicio del embarazo y durante la atención prenatal.

Compreender os fatores associados ao medo do parto em diferentes populações é essencial, pois os resultados podem variar significativamente devido a influências contextuais. Fatores como contextos sociais, etnias, religiões, crenças, percepções e legislações podem influenciar o olhar de homens e mulheres para o parto. Cada um desses elementos pode afetar as expectativas e experiências de homens e mulheres de maneiras distintas. Destaca-se a importância de abordagens culturalmente sensíveis e personalizadas no cuidado pré-natal e no apoio emocional. Analisar essas variáveis pode proporcionar uma compreensão mais profunda e abrangente, permitindo intervenções mais eficazes para mitigar o medo do parto e melhorar a experiência das gestantes. Com base nos achados desta pesquisa, acredita-se que estudos de intervenção para o medo do parto devem ser aprofundados para o desenvolvimento de estratégias preventivas e de suporte que promovam uma experiência mais positiva durante a gestação e o parto. Comprender los factores asociados con el miedo al parto en diferentes poblaciones es esencial, ya que los resultados pueden variar significativamente debido a influencias contextuales. Factores como contextos sociales, etnias, religiones, creencias, percepciones y legislación pueden influir en la visión de hombres y mujeres hacia el parto. Cada uno de estos elementos puede influir en las expectativas y experiencias de hombres y mujeres de diferentes maneras. Se destaca la importancia de enfoques culturalmente sensibles y personalizados en el cuidado prenatal y el apoyo emocional. Analizar estas variables puede proporcionar una comprensión más profunda y completa, permitiendo intervenciones más efectivas para mitigar el miedo al parto y mejorar la experiencia de las mujeres embarazadas. Según los hallazgos de esta investigación, se cree que los estudios de intervención por miedo al parto deberían desarrollarse para desarrollar estrategias preventivas y de apoyo que fomenten una experiencia más positiva durante el embarazo y el parto.

## Data Availability

Los conjuntos de datos relacionados con este artículo estarán disponibles previa solicitud al autor correspondiente.
